# Toxicity Study and Binding Analysis of Newly Synthesized Antifungal *N*-(4-aryl/cyclohexyl)-2-(pyridine-4-yl carbonyl) hydrazinecarbothioamide Derivative with Bovine Serum Albumin

**DOI:** 10.3390/ijms24054942

**Published:** 2023-03-03

**Authors:** Tanveer A. Wani, Ahmed H. Bakheit, Seema Zargar, Nojood Altwaijry, Mashooq Ahmad Bhat, Hamad M. Alkahtani, Lamees S. Al-Rasheed

**Affiliations:** 1 Department of Pharmaceutical Chemistry, College of Pharmacy, King Saud University, P.O. Box 2457, Riyadh 11451, Saudi Arabia; 2 Department of Biochemistry, College of Science, King Saud University, P.O. Box 22452, Riyadh 11451, Saudi Arabia

**Keywords:** BSA–ligand interaction, quenching mechanism, molecular docking, cytotoxicity, FRET, fluorescence

## Abstract

The presence of the p-aryl/cyclohexyl ring in the *N*-(4-aryl/cyclohexyl)-2-(pyridine-4-yl carbonyl) hydrazine carbothioamide derivative (2C) is reported to enhance the antifungal properties when compared to those of itraconazole. Serum albumins present in plasma bind and transport ligands, including pharmaceuticals. This study explored 2C interactions with BSA using spectroscopic methods such as fluorescence and UV-visible spectroscopy. In order to acquire a deeper comprehension of how BSA interacts with binding pockets, a molecular docking study was carried out. The fluorescence of BSA was quenched by 2C via a static quenching mechanism since a decrease in quenching constants was observed from 1.27 × 10^5^ to 1.14 × 10^5^. Thermodynamic parameters indicated hydrogen and van der Waals forces responsible for the BSA–2C complex formation with binding constants ranging between 2.91 × 10^5^ and 1.29 × 10^5^, which suggest a strong binding interaction. Site marker studies displayed that 2C binds to BSA’s subdomains IIA and IIIA. Molecular docking studies were conducted to further comprehend the molecular mechanism of the BSA–2C interaction. The toxicity of 2C was predicted by Derek Nexus software. Human and mammalian carcinogenicity and skin sensitivity predictions were associated with a reasoning level of equivocal, inferring 2C to be a potential drug candidate.

## 1. Introduction 

Invasive fungal infections are considered life-threatening infections [1]. They act as a major contributor to patient mortality and morbidity, particularly among those with weakened immune systems or who are hospitalized for other severe health conditions. Most of these infections occur due to *Candida* spp., with around 50% because of *Candida albicans* [2]. These fungi are liable for different types of sickness, from shallow diseases of the mucosal surfaces or skin to fundamental contaminations, which, by and large, is hazardous [2,3].

Generally, for therapy of any infection with *Candida* spp., amphotericin and azole drugs are utilized. However, these are not considered to fulfill clinical necessities because of their side effects, drug interactions, and the emergence of new resistant strains. Therefore, the development of safe medication against vulnerable strains is much needed [4].

**Figure 1:**
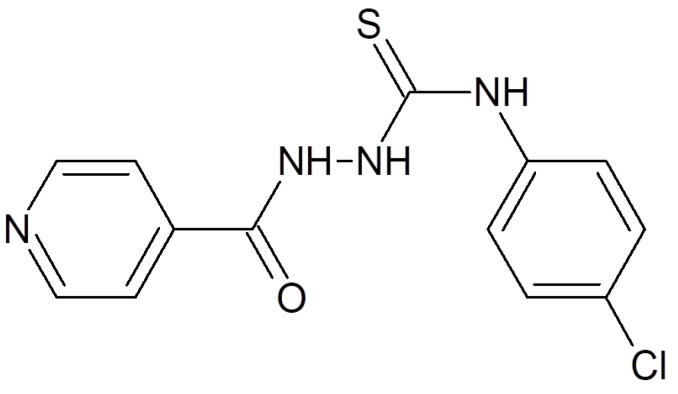
Chemical structure of a p-chloro derivative of *N*-(4-aryl/cyclohexyl)-2-(pyridine-4-yl carbonyl) hydrazine carbothioamide.

The existing literature revealed that some aryl thiosemicarbazones display an anti-*Candida* activity [5,6,7]. In the quest to develop novel antifungal drugs from the class of thiosemicarbazide derivatives against *Candida* spp., eighteen *N*-(4-aryl/cyclohexyl)-2-(pyridine-4-yl carbonyl) hydrazine carbothioamide derivatives were synthesized [8]. The synthesized compounds were tested with isolates of *Candida* spp., and the results were compared to itraconazole [8]. The p-chloro (2C) substitution at the phenyl ring of thiosemicarbazide improved the anti-*Candida* activity and was highly effective against *Candida albicans* ATCC 66027, *Candida* spp. 12,810 (blood), and *Candida* spp. 178 (HVS) [8] (Figure 1).

Ligands interact with proteins in the systemic circulation, and serum albumin is the most abundant protein in the blood. It maintains consistent osmotic pressure and transports biomolecules such as hormones, unsaturated fats, and medications throughout the body. After binding to serum albumin, ligands are delivered to their sites of action [9,10,11,12]. The interaction between ligands and proteins has a substantial effect on the pharmacodynamics and pharmacokinetics of the ligands. Strong or poor binding of ligands to serum albumin influences the free ligand concentration in the systemic circulation. A poor binding may result in a high concentration of free drug and a rapid rate of excretion. In contrast, a highly strong binding may reduce the amount of medicine that is freely available, resulting in a subtherapeutic response and a longer elimination period [13,14].

Since the bovine serum albumin (BSA) and the HSA (human serum albumin) are similar in structure and BSA is readily available, the HSA is substituted with BSA in the protein–ligand interaction studies. The BSA–2C interaction was studied using fluorescence and UV absorbance spectroscopy. The parameters investigated in the study were quenching mechanisms, binding constant, identification of the binding site, thermodynamics, synchronous fluorescence spectroscopy, and Forster resonance energy transfer (FRET). Molecular docking for the BSA–2C system was performed to verify the experimental results. This study will be helpful in assessing the binding mechanism of BSA–2C that might be valuable for preclinical pharmacokinetic considerations.

In vitro research is a tool in the early assessment studies of drugs and provides a less expensive and ethically feasible option against the testing of medications in animals. Consequently, 2C was assessed for its potential toxicity utilizing the in silico technique. The toxicity of 2C was assessed with the software Lhasa nexus 2.2.2 (2018), Derek Nexus v 6.0.1 [9].

## 2. Results and Discussion

### 2.1. Synthesis

Following the methodology laid out by Bhat et al., the synthesis of the isoniazid (INH) derivative 2C was completed in a single step [8]. To summarize, isoniazid was combined with phenyl/cyclohexyl isothiocyanate in the presence of 100% ethanol to produce *N*-(4-aryl/cyclohexyl)-2-(pyridin-4-yl carbonyl) hydrazine carbothioamide. This compound has the chemical formula C13H11N4OSCl and the molecular weight 306.77 (Figure 1).

### 2.2. Fluorescence Quenching

#### 2.2.1. Stern–Volmer Analysis

The presence of Trp residues in BSA is primarily responsible for its fluorescence properties. At a given concentration of BSA, fluorescence spectra were recorded at an excitation of 280 nm and emission of 300–500 nm, while the 2C concentration fluctuated from 0.0 to 18.0 M.

**Figure 2:**
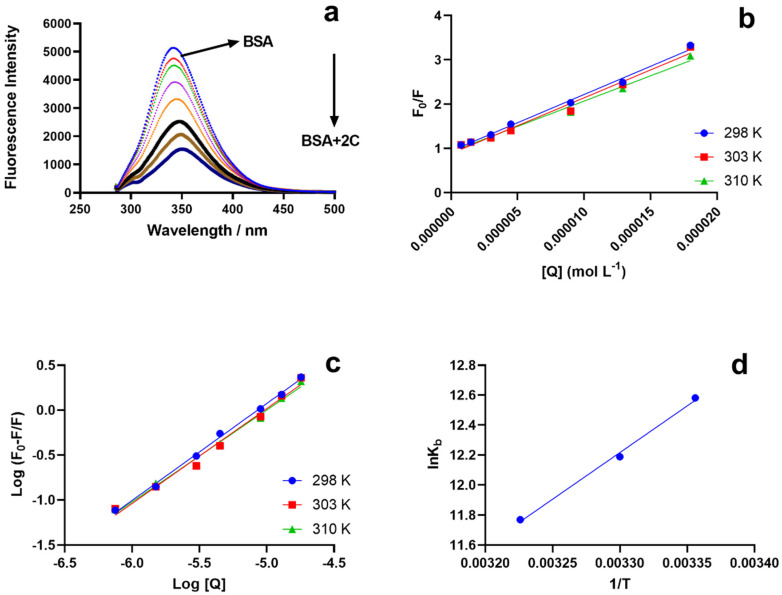
(**a**), BSA’s steady-state fluorescence emission spectra acquired with and without varying amounts of 2C. Fluorescence quenching of BSA during titration with 2C is depicted by the black arrow. (**b**) Illustration of the Stern–Volmer plot for the BSA–2C interaction at 298 K, 301 K, and 310 K. (**c**) Binding constants for the BSA–2C system as a function of temperature (295 K, 300 K, 310 K). (**d**) Van ’t Hoff plot, lnK vs. 1/T. In all the studies (**a**–**d**), 1.5 μM of BSA was used and titrated with 2C (0, 0.75, 1.5, 3.0, 4.5, 9.0, 13.0, 18.0 μM).

The fluorescence intensity decreased as the concentration of 2C increased (Figure 2a). In the BSA–2C interaction, a redshift of 7 nm was seen in the emission wavelength, demonstrating that amino acids in a polar environment were more exposed to solvents. In the case of static quenching, the Stern–Volmer (Ksv) result dropped at high temperatures, whereas it increased in the case of dynamic quenching. The drop in Ksv for the BSA–2C system implies that the quenching results from the creation of a static complex rather than a dynamic one. Figure 2b depicts the Stern–Volmer plot (Equation (1)) for the BSA–2C system at the three studied temperatures. The Ksv quenching constants at various temperatures (298, 301, and 310 K) are shown in Table 1. The biomolecular quenching constants, k_q_, were also determined for the BSA–2C system (Equation (2)). In the absence of a quencher, the lifespan of the fluorophore, τ_0_, was 6 ns for BSA. The calculated k_q_ values exceeded the maximum diffusion collision quenching rate constant of 2.0 × 10^10^ LS^−1^ mol^−1^ [15,16] (Table 1). The high maximum scatter collision quenching constant values further suggest the formation of a ground-state complex and static quenching mechanism involved in the BSA–2C interaction.

#### 2.2.2. Binding Constants

Quenching experiments and complex formation between BSA and 2C provide strong evidence for the binding of 2C to BSA. By fitting a double logarithmic regression curve, we were able to calculate the binding affinity, K_b_, and the cooperativity factor, n, of 2C to BSA (Hill equation, Equation (2)). In order to determine the binding constant, we used the slope of the line connecting log (F_0_ − F)/F and the log molar concentration of 2C (Figure 2c). Constants for the BSA–2C binding system are listed in Table 2. Increasing temperatures resulted in smaller binding constants, and the data suggest a very strong binding interaction (>10^5^) between BSA and 2C. However, no binding cooperativity was observed since “n” was almost equal to 1, which means there was no influence on the binding cooperativity. Further, the binding cooperativity remained unchanged at all the studied temperatures [17,18].

#### 2.2.3. Thermodynamic Interactions

The signs and values of the entropy and enthalpy parameters identify the type of interactions. The enthalpy change (∆H°), the entropic change (∆S°), and the free energy change (∆G°) were calculated from Equations (2) and (3). Van der Waals interactions or hydrogen bonding between BSA and 2C are indicated by the Van ‘t Hoff plot’s negative values for entropy, enthalpy, and free energy (Figure 2d) (Table 2) [19,20,21,22,23].

#### 2.2.4. Binding Site Identification

**Figure 3:**
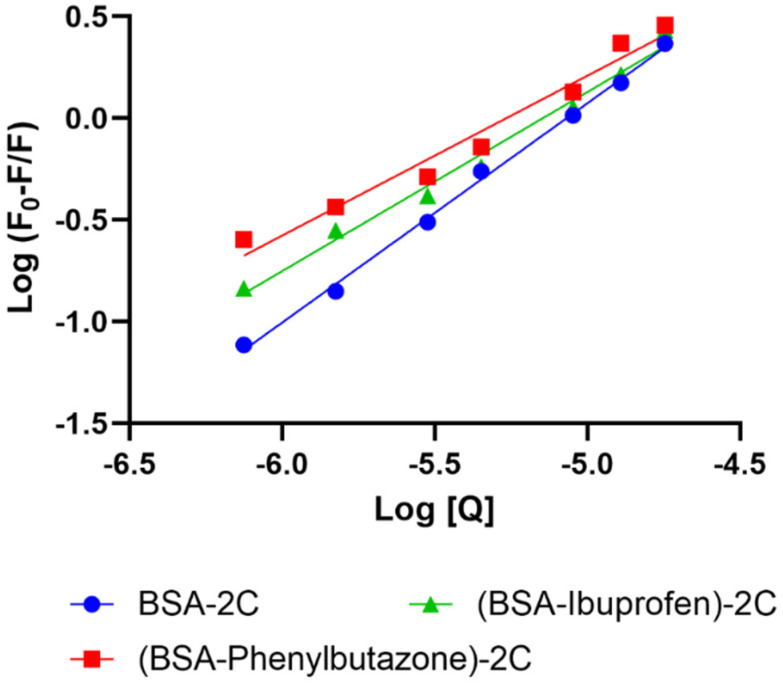
Plot for the BSA–2C system with phenylbutazone and ibuprofen as site markers, measured at 298 K.

Using displacement studies using phenylbutazone (PHB) and ibuprofen (IBP) as site markers for Site I and Site II of BSA, respectively, the binding site for 2C on BSA was determined. For the BSA–2C system, binding constants with the presence of PHB (2.5 μM) and IBP (2.5 μM) were investigated and compared to binding constants in which they were absent. In the presence of PHB and IBP, the binding constants for the BSA–2C system were significantly reduced from 2.91 × 10^5^ to 2.11 × 10^4^ and 1.34 × 10^4^, respectively. Furthermore, when both site markers were present, the binding constants dropped roughly equally. As a result, we speculate that 2C may bind to both locations (Site I and Site II of BSA) (Figure 3) [9]. There is evidence that ibuprofen may bind to site 1, although with a rather low affinity [24]. At high ibuprofen concentrations ranging from 0 to 600 μg mL^−1^, nifedipine was dislodged from its binding sites by ibuprofen [25]. At the levels utilized in our experiment, however, ibuprofen has been efficiently used as a site marker for Site II [26].

### 2.3. UV Absorption Spectroscopy

**Figure 4:**
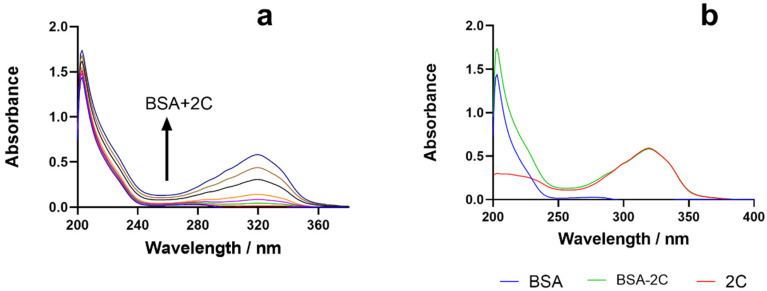
(**a**) BSA’s UV-absorption spectra in the presence of 2C (0–18.00) μM. (**b**) UV absorption spectra of BSA, BSA–2C, and 2C.

Comparing the absorption spectra of BSA and BSA–2C (Figure 2), in the presence of 2C, a shift in the absorption spectrum of BSA was seen at 280 nm and 210 nm. In the case of dynamic quenching, the absorption spectra should be unaffected by ligand concentration changes, however in the case of static quenching, a complex forms between the BSA and the ligand, causing the UV spectra to shift as the ligand concentration changes [14]. The 280 nm absorption spectrum of BSA is related to aromatic amino acid transitions, while the 210 nm spectrum reflects the conformational framework of BSA. The interaction of BSA with 2C resulted in a shift in the absorption spectrum (Figure 4a). With the addition of 2C, the absorbance of BSA increased, indicating the formation of a 2C–BSA combination at 203 nm. With the addition of 2C, a little shift was also noticed in the absorption spectrum of BSA at 280 nm [27,28,29]. Comparing the absorption spectra of BSA–2C to those of BSA and 2C, an increase in absorption at 280 nm was seen in the BSA–2C system, demonstrating the existence of a static quenching mechanism between BSA and 2C (Figure 4b).

### 2.4. Forster Resonance Energy Transfer (FRET) between BSA and 2C

**Figure 5:**
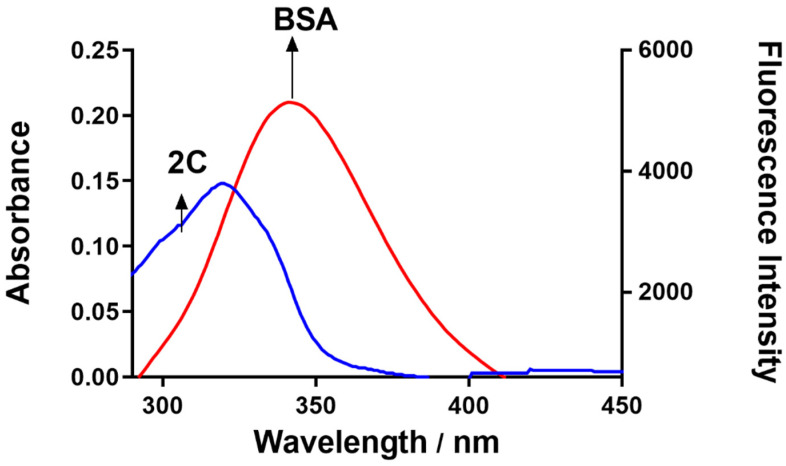
FRET study for BSA and 2C at an equimolar concentration of 1.5 μM.

Based on Equations (6)–(8), we can determine that BSA had a FRET of 0.118 under conditions where K^2^ = 2/3, the refractive index of the medium = 1.336, and D = 0.118. Table 3 displays the results of the calculations. Having a “r” value of 2.90 indicates a highly significant degree of closeness between the donor and the acceptor. It also shows that there is a chance of non-radiative energy transfer from BSA to 2C, as the distance between the donor and acceptor was smaller than 8 nm (Figure 5) [30].

### 2.5. In Vitro Toxicity Prediction of 2C

Derek Nexus software was used to conduct an in silico toxicity analysis to assess the toxicity of 2C. The hydrazine moiety of 2C was shown to be hazardous to humans, animals, and microbes. It displayed alerts for skin hypersensitivity, kidney and liver damage, mitochondrial dysfunction, teratogenicity, and possible carcinogenicity. The toxicity response was classified as equivocal and plausible (Supplementary File S1). The 2C showed equivocal toxicity for mitochondrial dysfunction, nephrotoxicity, and skin sensitization, and plausible toxicity for teratogenicity, carcinogenicity, and hepatotoxicity concerning the endpoint alerts hydrazine, halogenated benzene, and hydrazine precursor in humans and other mammals. The presence of a structural moiety consisting of hydrazine indicated a significant potential for carcinogenic action.

Hydrazine was most likely activated metabolically by the process of *N*-hydroxylation, or through the formation of free radicals [31]. Additionally, hydrazines and hydrazides could potentially cause protein deglycation with the consequent release of glycated hydrazone adducts [32], and they have the potential to serve as haptens [22]. The in-computer analysis of 2C’s toxicity is useful for planning future research and development of the 2C as a suitable drug candidate. The promising in silico toxicity profile of the investigational drug and its therapeutic potential will be beneficial in developing other studies for further development of the drug.

### 2.6. Synchronous Fluorescence Studies

**Figure 6:**
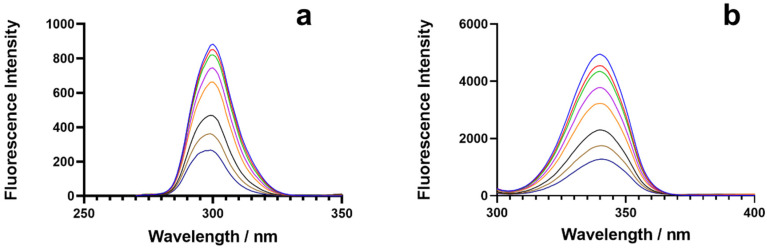
Synchronous fluorescence spectroscopic data of BSA at 298 K at (**a**) ∆λ = 15 nm and (**b**) ∆λ = 60 nm in the presence of DHP.

As a result of a ligand’s presence, the protein’s environment undergoes changes, and the SFS can be utilized to better understand these changes [12,22]. The tryptophan and tyrosine residues in BSA are responsible for its intrinsic fluorescence, and a shift in the emission they give off indicates a shift in the protein’s immediate surroundings. With ∆λ, 15 nm for the tyrosine residue and 60 nm for the tryptophan residue, the SFS was successfully recorded. Tyrosine and tryptophan both saw a 1 nm shift in their surroundings at ∆λ (15 nm) and no change at ∆λ (60 nm) due to 2C (Figure 6) [33].

### 2.7. Molecular Docking

**Figure 7:**
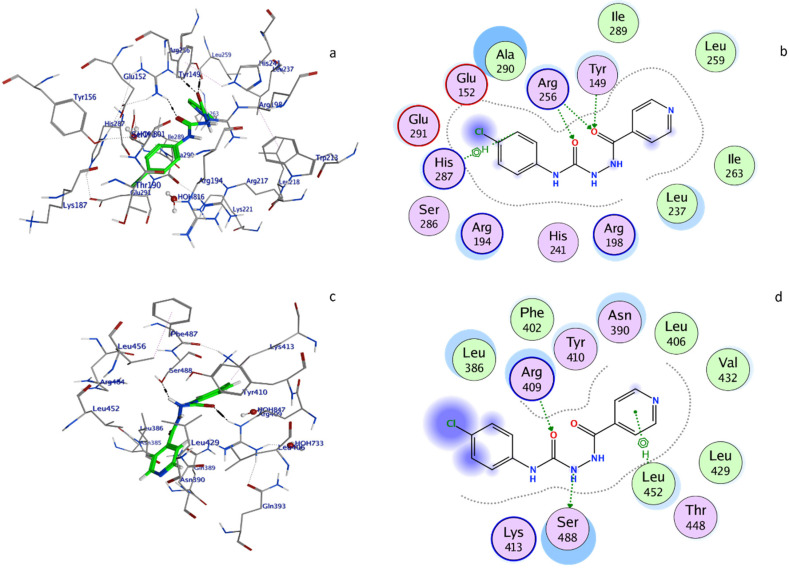
BSA–2C system in three-dimensional conformation: (**a**) Site I and (**c**) Site II. Amino acid structure surrounding 2C in a two-dimensional conformation around Site I (**b**) and Site II (**d**).

**Figure 8:**
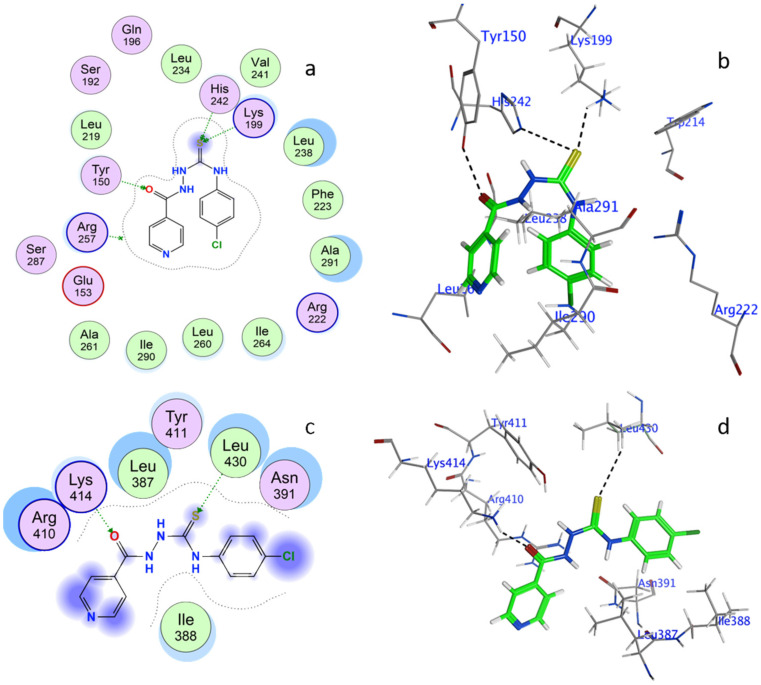
Has–2C system in three-dimensional conformation: (**a**) Site I and (**c**) Site II. Amino acid structure surrounding 2C in a two-dimensional conformation around Site I (**b**) and Site II (**d**).

The molecular docking was used to retrieve the information about BSA–2C interactions [34,35]. The 2C protein docked with BSA protein, and the results were −5.62 and −5.22 Kcal/mol. When interacting with the amino acids in the BSA–2C interaction, the 2C positioned itself to engage with them at Site I in subdomain IIA and Site II in subdomain IIIA. As is clear from the two-dimensional figure that the following amino acids surround 2C at Site I: HIS287, GLU291, GLU152, ALA290, ARG256, TYR149, ILE289, LEU259, ILE263, LEU237, ARG198, HIS241, ARG194, and SER286, and LEU286, PHE402, ARG409, TYR410, ASN390, LEU406, VAL432, LEU429, THR448, LEU452, SER488, and LYS413 amino acids at Site II. Based on the docking data, it was confirmed that both the subdomains IIA/IIIA of BSA were engaged in the interaction, as suggested by the binding site displacement experiments. Further, three hydrogen bonds, one with TYR149 (O19-OH TYR149, 2.76A) and two with ARG256 (O19-NE ARG256, 3.26A, and O31-NH2 ARG256, 2.92A), and a pi-hydrogen bond with HIS287 (C22-5-ring HIS287, 4.61A), were observed at Site I of the subdomain IIA interaction. Similarly, there were two hydrogen bonds by SER488 (N13-OG SER488, 3.02A) and ARG409 (O31-NE ARG409, 2.80A). In addition, a pi-hydrogen bond with LEU452 (6-ring-CD1LEU452, 4.72A) was noticed at site II under subdomain IIIA as well. Hydrogen bonding is likely involved in the BSA–2C interaction, as suggested by the molecular docking results, which are consistent with the experimental results. Furthermore, molecular docking analyses showed that 2C binds to both subdomains IIA and IIIA of BSA (Figure 7). A certain degree of dissimilarity occurred between BSA and HSA structures, and therefore the interaction efficiency and binding modes were compared between the proteins on its interaction with 2C. Three hydrogen bonds were observed between O16-OH TYR150, 2.64A, S20-NZ LYS199, 3.14A, and S20-NE2 HIS242, 3.82A, at Site I, whereas two hydrogen bonds were observed between O16-NZ LYS414, 2.98A, and S20-CD2 LEU430, 4.27A, at Site II. Molecular docking analysis showed that 2C binds to both Site I and Site II of HSA (Figure 8).

**Figure 9:**
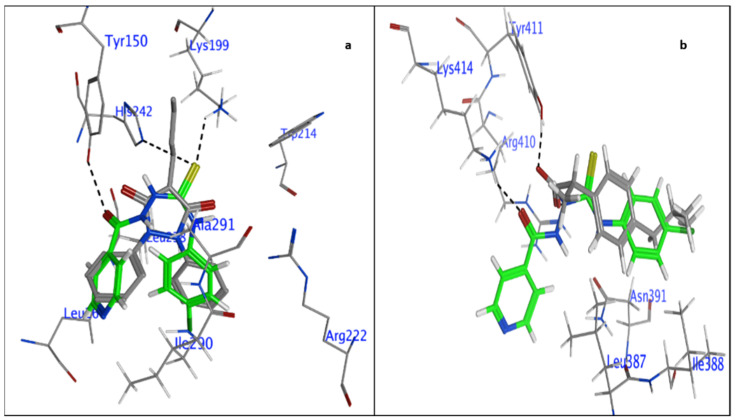
The superimposed conformation of (**a**) 2C with HSA–phenylbutazone and (**b**) HSA–ibuprofen.

In addition, the binding sites were verified using proteins 2bxc and 2bxg, where phenylbutazone and ibuprofen were co-crystalized with HSA. The superimposed conformation of HSA–phenylbutazone with 2C and HSA–ibuprofen with 2C are presented in Figure 9. The docking conformation and validation of 2C, PHB, and IBP are presented in Figure S1A,B (Supplementary File S2).

## 3. Materials and Methods

### 3.1. Chemicals

The synthesis of compound 2C was performed by Bhat et al. [8]. The BSA (fatty acid-free) was procured from Sigma Aldrich (St. Louis, MO, USA). Ibuprofen and phenylbutazone, used as site markers, were obtained through National Scientific Company (Riyadh, KSA).

### 3.2. Sample Preparation

The BSA stock solution was made using phosphate-buffered saline (pH 7.4). To prepare the working solutions, acetonitrile was used to dissolve the 6M stock of 2C, and then PBS (pH 7.4) was added to adjust the concentrations to the required amounts.

### 3.3. Fluorescence Spectroscopy

The fluorescence was measured using a JASCO Model FP-8200 fluoro-spectrophotometer. The spectra were taken at 298, 303, and 310 K, with a constant BSA concentration of 1.5 μM and a range of ligand concentrations from 0.00 to 18.00 μM, as follows: 0, 0.75 × 10^−6^, 1.5 × 10^−6^, 3.0 × 10^−6^, 4.5 × 10^−6^, 9.0 × 10^−6^, 13.0 × 10^−6^, and 18.0 × 10^−5^. The excitation wavelength was set to 280 nm, whereas the emission wavelength was 300–500 nm. Ligands quench the BSA fluorescence, and data obtained from the BSA–2C interaction were evaluated with the Stern–Volmer equation [36]:

(1) $$\frac{F_{0}}{F}=1+K_{\mathrm{sv}}\left[Q\right]$$

where F and F_0_ are the fluorescence intensity (F.I.) of BSA with and without 2C, respectively. K_sv_ and [Q] represent the Stern–Volmer quenching constant and the quencher concentration, respectively. Further, bimolecular rate constant k_q_ values were also evaluated to determine the quenching mechanism [36,37]:

(2) $$k_{q}=K_{\mathrm{sv}}/\tau_{0}$$

where τ is the excited state lifetime of BSA without 2C. The τ_0_ has been determined to be 6 ns for BSA [38,39].

The double-log regression plot expresses the molecular equilibrium, and the binding constants “K_b_” can be determined for the BSA–2C system with the equation:

(3) $$\log\frac{\left(F_{0}-F\right)}{F}={\log\;K}_{b}+n\;\log\left[Q\right]$$

where n is the Hill coefficient, which depicts the extent of cooperation in the protein–ligand binding.

Synchronous fluorescence spectroscopy (SFS) can be used to examine the minute changes in the surroundings of the amino acid residues tryptophan and tyrosine. Characteristics of tryptophan and tyrosine residues were captured in the BSA–2C SFS spectra at ∆λ = 60 and 15 nm, respectively.

### 3.4. Thermodynamics

The degree and specificity of interactions between partners in complex formation determine the enthalpic contribution to free energy (G°). The BSA–2C interactions might include ionic, electrostatic, van der Waals, and hydrogen bonds. The types of signs and magnitude of the calculated parameters ∆G°, ∆H° (enthalpy), and ∆S° (entropy) provide evidence of the interactions involved.

(4) $${\mathrm{lnK}}_{b}=-\frac{\Delta H^{{}^{\circ}}}{\mathrm{RT}}+\frac{\Delta S^{{}^{\circ}}}{R}$$

(5) $$\Delta G^{{}^{\circ}}=\Delta H^{{}^{\circ}}-T\Delta S^{{}^{\circ}}$$

### 3.5. Site Markers’ Elucidation and Microenvironmental Changes

The binding site of 2C on BSA was determined by competitive binding of site-specific markers phenylbutazone and ibuprofen with 2C at room temperature.

### 3.6. Forster Resonance Energy Transfer (FRET)

FRET can occur when the emission and absorption spectra of the donor and acceptor molecules coincide. When the distance between the 2C (acceptor) and BSA (donor) fluorophores is less than 10 nm, the amount of energy transferred from the 2C to BSA may be quantified. Equimolar concentrations (1.5 μM) of BSA and 2C were analyzed for their fluorescence emission spectra and the absorption spectra. The BSA was used for emission, whereas 2C was used for the absorption spectra. The FRET is calculated as:

(6) $$E=1-\frac{F_{0}}{F}=\frac{R_{0}^{6}}{R_{0}^{6}+r^{6}}$$

where E represents energy transfer efficiency, and r and R_0_ represent donor and acceptor critical binding distances, respectively.

At 50% energy transfer:

(7) $$R_{0}^{6}=8.79\times 10^{-25}K^{2}\phi_{D}n^{-4}J$$

where K^2^ = dipole orientation, and n and ϕ are the medium’s refractive index and donor’s quantum yield, respectively.

The overlap in the spectra (emission and absorption) is given as:

(8) $$J\left(\lambda \right)=\frac{\sum F\left(\lambda \right)\epsilon \left(\lambda \right)\lambda^{4}\Delta \lambda }{\sum \left(\lambda \right)\Delta \lambda }$$

where J is the extent of overlap, F(λ) is the donor emission spectrum, and ϵ(λ) is the molar absorptivity coefficient of the acceptor.

### 3.7. UV-Vis Absorption Spectra

Absorption spectra were recorded using a Shimadzu UV-1800 (Kyoto, Japan) spectrophotometer. Spectra of BSA were obtained at a range of 2C concentrations (0.00–18 μM). The content of BSA was held constant in all samples at 1.5 μM, and all measurements were performed at 298 K.

### 3.8. Toxicity Predictions

Lhasa nexus 2.2.2 (2018), Derek Nexus v 6.0.1 software was used to determine the toxicity of 2C. The software assigns probabilities and confidence intervals for each toxicity level, from certain and reasonable, to equivocal and doubtful, to improbable and impossible. The software was used to forecast the toxicity of 2C.

### 3.9. Molecular Docking

The molecular docking in silico binding interactions were conducted using the Molecular Operating Environment (MOE). The structure of 2C was drawn in Marvin version 21.19.0, ChemAxon (https://www.chemaxon.com (accessed on 15 January 2023)), and converted to .pdb* format in the MOE software. The PDB structure of BSA (PDB ID: 6qs9) was downloaded from the protein data bank (http://www.rcsb.org/pdb (accessed on 15 January 2023)). The default field force parameters were used for the study. The triangular matcher was set with default parameters, with scoring function 1 as London dG and function 2 as GBVI/WSA dG.

## 4. Conclusions

In this research, we looked at how 2C reacted with BSA. According to the fluorescence analyses, the BSA–2C interaction was governed by a static quenching mechanism. UV-visible analyses confirmed that 2C formed a complex with BSA. Site I in subdomain IIA and Site II in subdomain IIIA of BSA were identified as binding sites for the 2C. Thermodynamic studies pointed to the importance of van der Waals forces and hydrogen bonding in the interaction. In addition, the study’s findings on 2C’s toxicity in the screening phase, where 2C shows promise as an anti-fungal drug candidate, are extremely important.

## Figures and Tables

**Table 1:** The biomolecular quenching constant, k_q_, and the quenching constant, Ksv, for the BSA–2C interaction.

T(K)	R	Ksv ± SD × 10^5^ (M^−1^)	K_q_ × 10^13^ (M^−1^S^−1^)
298	0.9942	1.275 ± 0.19	2.125
303	0.9820	1.259 ± 0.12	2.098
310	0.9876	1.143 ± 0.16	1.905

**Table 2:** Binding and thermodynamic parameters for BSA–2C.

T(K)	K_b_ ± SD	n	∆G° ± SD (kJ mol^−1^)	∆H° ± SD (kJ mol^−1^)	∆S° ± SD (J mol^−1^·K^−1^)
298	(2.91 ± 0.27) × 10^5^	1.0782	−33.40	−70.596	−124.80
303	(1.96 ± 0.20) × 10^5^	1.0554	−32.78		
310	(1.29 ± 0.19) × 10^5^	1.0221	−32.28		

**Table 3:** Energy transfer for BSA and 2C at equimolar concentrations (1.5 × 10^−6^ M).

	J (cm^3^ mol^−1^)	E	R_0_ (nm)	r (nm)
BSA–2C system	1.48 × 10^−14^	0.354	2.62	2.90

## Data Availability

Data will be available on request to corresponding author.

## Supplementary Materials

The following supporting information can be downloaded at: https://www.mdpi.com/article/10.3390/ijms24054942/s1.
