# Supplementary material for: Toxicity Study and Binding Analysis of Newly Synthesized Antifungal N-(4-aryl/cyclohexyl)-2-(pyridine-4-yl carbonyl) hydrazinecarbothioamide Derivative with Bovine Serum Albumin
Source: Int J Mol Sci. 2023 Mar 3;24(5):4942. doi: 10.3390/ijms24054942 (PMC10002925; doi:10.3390/ijms24054942)
Supplement: Supplementary file 1 [file ijms-24-04942-s001.zip › Supplementary File S1.pdf]

Derek Nexus Report

Report Information

Author	Report date	Prediction date	Program version
abakheit	20 September 2020 10:36:33	20 September 2020 10:35:57	Derek Nexus: 6.0.1, Nexus: 2.2.2

Processing Options

Selected Species
bacterium, human, mammal

Selected Knowledge Base(s)
Derek KB 2018 1.1

Reasoning Level
At least EQUIVOCAL

Perceive tautomers
Yes

Perceive mixtures
Yes

Match alerts without rules
No

Show Open likelihood
No

Show Negative Predictions
Yes

Show Rapid Prototypes
Yes

Filter nearest neighbours on misclassified features
Yes

Submitted Compound - Structure-1

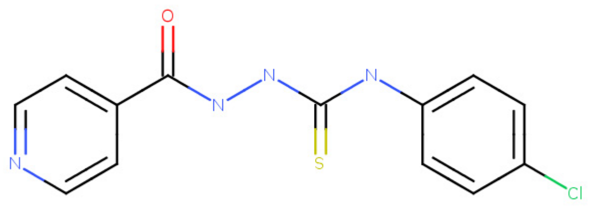

Smiles:
C=1([H])C([H])=NC([H])=C([H])C=1C(N([H])N([H])C(N([H])C=2C(=C(C(Cl)=C(C=2[H])[H])[H])[H])=S)=O

**Exact Mol Mass**	306.0342
**Average Mol Mass**	306.77 (Source: User Supplied)
**Log Kp**	-3.97 (Source: User Supplied)
**Log P**	0.87 (Source: User Supplied)

Predictions

Knowledge Base: Derek KB 2018 1.1

Version	Last Modified Date	Certified by
1.1	23/11/2017 12:38:05	Lhasa Limited, Leeds, Yorkshire, UK

Reasoning Summary

◆ **Carcinogenicity in human is PLAUSIBLE**
- Alert matched: 114 Hydrazine

◆ **Carcinogenicity in mammal is PLAUSIBLE**
- Alert matched: 114 Hydrazine

◆ **Hepatotoxicity in human is PLAUSIBLE**
- Alert matched: 615 Hydrazine

◆ **Hepatotoxicity in mammal is PLAUSIBLE**
- Alert matched: 615 Hydrazine

◆ **Mitochondrial dysfunction in human is EQUIVOCAL**
- Alert matched: RapidPrototype108 Hydrazine

◆ **Mitochondrial dysfunction in mammal is EQUIVOCAL**
- Alert matched: RapidPrototype108 Hydrazine

◆ **Mutagenicity in vitro in bacterium is INACTIVE**

- Contains unclassified features

◆ **Nephrotoxicity in human is EQUIVOCAL**
- Alert matched: RapidPrototype035 Halogenated benzene
- Alert matched: RapidPrototype036 Hydrazine

◆ **Nephrotoxicity in mammal is EQUIVOCAL**
- Alert matched: RapidPrototype035 Halogenated benzene
- Alert matched: RapidPrototype036 Hydrazine

◆ **Skin sensitisation in human is EQUIVOCAL**
- Alert matched: 448 Hydrazine or precursor

◆ **Skin sensitisation in mammal is EQUIVOCAL**
- Alert matched: 448 Hydrazine or precursor

◆ **Teratogenicity in human is PLAUSIBLE**
- Alert matched: 605 Hydrazine

◆ **Teratogenicity in mammal is PLAUSIBLE**
- Alert matched: 605 Hydrazine

Endpoints not firing any alerts at the selected reasoning level (54)

5alpha-Reductase inhibition
Adrenal gland toxicity
alpha-2-mu-Globulin nephropathy
Anaphylaxis
Androgen receptor modulation
Bladder disorders
Bladder urothelial hyperplasia
Blood in urine
Bone marrow toxicity
Bradycardia
Cardiotoxicity
Cerebral oedema
Chloracne
Cholinesterase inhibition
Chromosome damage in vitro
Chromosome damage in vivo
Cumulative effect on white cell count and immunology
Cyanide-type effects
Developmental toxicity
Glucocorticoid receptor agonism
HERG channel inhibition in vitro
High acute toxicity
Irritation (of the eye)
Irritation (of the gastrointestinal tract)
Irritation (of the respiratory tract)
Irritation (of the skin)
Kidney disorders
Kidney function-related toxicity
Lachrymation
Methaemoglobinaemia
Mutagenicity in vivo
Neurotoxicity
Non-specific genotoxicity in vitro
Non-specific genotoxicity in vivo
Occupational asthma
Ocular toxicity
Oestrogen receptor modulation
Oestrogenicity
Peroxisome proliferation
Phospholipidosis
Photo-induced chromosome damage in vitro
Photo-induced non-specific genotoxicity in vitro
Photo-induced non-specific genotoxicity in vivo
Photoallergenicity
Photocarcinogenicity
Photomutagenicity in vitro
Phototoxicity
Pulmonary toxicity
Respiratory sensitisation
Splenotoxicity
Testicular toxicity
Thyroid toxicity
Uncoupler of oxidative phosphorylation
Urolithiasis

Alert Descriptions

Alert: 114 Hydrazine (from KB: Derek KB 2018 1.1)

Alert Description Image

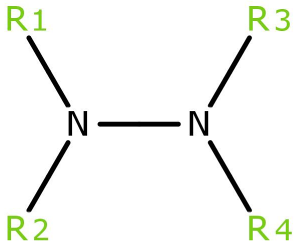

R1-R4 = C, H
Of which at least two
must be hydrogens

Match with query compound

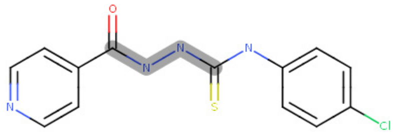

Comments

Many hydrazines are known to be carcinogenic. The presence of this structural moiety gives a high possibility of carcinogenic activity. Metabolic activation is thought to be necessary, probably via N-hydroxylation.

Validation comments

Carcinogenicity

The alert has demonstrated the following predictive performance:

1) CPDB data set: 57 compounds activate this alert of which 44 are reported positive (positive predictivity = 77%)
2) ToxRefDB data set: 2 compounds activate this alert of which 1 is reported positive (positive predictivity = 50%)
3) Brambilla data set: 5 compounds activate this alert of which 4 are reported positive (positive predictivity = 80%)

1) A collection of carcinogenicity data for 1547 compounds from the following source: Carcinogenic Potency Database (CPDB) downloaded from DSSTox (version 5d, revised 20 November 2008), available at "http://www.epa.gov/NCCT/dsstox/sdf_cpdbas.html".

2) A collection of carcinogenicity data for 337 compounds from the following source: Toxicity Reference Database (ToxRefDB) Chronic & Cancer Endpoints data (downloaded 21 August 2012 from EPA website), which is no longer available. This data set was derived from that described in the following reference: Martin MT, Judson RS, Reif DM, Kavlock RJ and Dix DJ. Profiling chemicals based on chronic toxicity results from the U.S. EPA ToxRef database. Environmental Health Perspective, 2009, 117, 3, available at "http://dx.doi.org/10.1289/ehp.0800074".

3) A collection of carcinogenicity data for 537 compounds derived from the following references: (i) Brambilla G and Martelli A. Update on genotoxicity and carcinogenicity testing of 472 marketed pharmaceuticals. Mutation Research, 2009, 681, 209-229, available at "http://dx.doi.org/10.1016/j.mrrev.2008.09.002"; (ii) Brambilla G, Mattioli F, Robbiano L and Martelli A. Update of carcinogenicity studies in animals and humans of 535 marketed

pharmaceuticals. Mutation Research, 2012, 750, 1-51, available at "http://dx.doi.org/10.1016/j.mrrev.2011.09.002".

In assessing predictive performance, it should be noted that:

- Mammalian carcinogenicity predictions in Derek associated with a reasoning level of equivocal or above have been considered positive;

- Predictions do not take into account (i) the tautomeric forms of compounds or (ii) the individual components of mixtures;

- The classification of compounds from the CPDB data set as active or inactive is based upon the "ActivityOutcome_CPDBAS_SingleCellCall" field as defined within DSSTox;

- The classification of compounds from the ToxRefDB data set as active or inactive is based upon an overall call assigned from the tumorigenicity summary information. Any active in a single species leads to an overall active call;

- The classification of compounds from the Brambilla data set as active or inactive is based upon carcinogenicity studies in mammals. Consistent reports of activity in a single species leads to an overall active call;

- Compounds in the data sets assigned responses other than active or inactive have been excluded from the analysis;

- No account has been taken of other carcinogenicity alerts which may also be present in some compounds;

- No comparison has been made between the species, strain, gender or route of administration dependency of positive experimental results and the expected profile which may be included in the comments for an alert;

- Information from the data sets may have been used previously as supporting evidence for the derivation of some alerts;

- Some compounds may be present in more than one of the data sets analysed.

Alert: 448 Hydrazine or precursor (from KB: Derek KB 2018 1.1)

Alert Description Image | **Match with query compound**

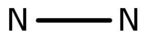

Nitrogen atoms cannot be aromatic or be attached to additional heteroatoms

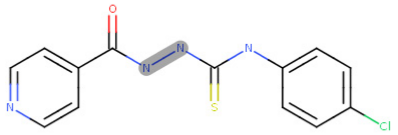

Comments

Skin sensitisation: murine local lymph node assay (LLNA), guinea pig maximisation test (GPMT), human patch test
Potential mechanism: Prehapten producing an electrophile, miscellaneous [Payne and Walsh]
Potential mechanism: Prehapten producing a free radical generator [Payne and Walsh]

Hydrazine and phenylhydrazine are classified as contact allergens (BgVV category A) [Kayser and Schlede] and positive human patch test results were reported for hydrazine and selected derivatives [Foulds and Koh, Pevny and Peter, Wheeler et al, Suzuki and Ohkido]. Conversely, some human patch test studies have reported negative results [Rothe]. Hydrazides such as carbonohydrazide showed positive activity in the GPMT [ECHA 2001], although the structural analogue adipohydrazide was negative in the LLNA [ECHA 2004]. Additionally, hydrazine derivatives such as phenylhydrazine are reported as irritant and corrosive agents [NIOSH], and it is possible that these compounds may give false positive responses in the human patch test and the GPMT.

Hydrazines and hydrazides could potentially cause protein deglycation with the consequent release of glycated hydrazone adducts [Kobayashi et al] which could act as haptens [Gould and Taylor]. Additionally, hydrazine based compounds can generate radical species in the presence of metal ions [Sinha and Mason] and this could explain why cases of contact dermatitis have been reported in people working in contact with metals [Wheeler et al]. This suggests that hydrazines can act as prehaptens in the presence of a metal ion.

The scope of this alert has been defined on the available data and putative mechanism of hydrazine and precursors. As a consequence of the scarcity of toxicological data from animal studies and the questionable human data, the likelihood level of predictions in mammals for alerting structures has been set as equivocal.

References

- Kayser D and Schlede E (editors). (2001)
  Chemikalien und Kontaktallergie: Eine Bewertende Zusammenstellung., *Chemikalien und Kontaktallergie: Eine Bewertende Zusammenstellung*, , All pages

- Payne MP and Walsh PT. (1994)
  Structure-activity relationships for skin sensitization potential: development of structural alerts for use in knowledge-based toxicity prediction systems., *Journal of Chemical Information and Computer Sciences*, 34 , 154-161
  DOI: 10.1021/ci00017a019
- Wheeler C Jr, Penn SR and Cawley EP. (1965)
  Dermatitis from hydrazine hydrobromide solder flux., *Archives of Dermatology*, 91 , 235-239
  DOI: 10.1001/archderm.1965.01600090043008
- Sinha BK and Mason RP. (2014)
  Biotransformation of hydrazine derivatives in the mechanism of toxicity., *Journal of Drug Metabolism and Toxicology*, 5 , 168
  DOI: 10.4172/2157-7609.1000168
- Gould JC and Taylor S. (2011)
  Hazard identification of strong dermal sensitizers., *Toxicology Mechanisms and Methods*, 21 , 86-92
  DOI: 10.3109/15376516.2010.484622
- Kobayashi K, Yoshimoto K, Hirauchi K and Uchida K. (1993)
  Deglycation of glycated proteins with hydrazine analogues., *Life Sciences*, 53 , 291-295
  DOI: 10.1016/0024-3205(93)90681-R
- Rothe A. (1988)
  Contact dermatitis from N-(alpha-chlorobenzylidene)phenylhydrazine., *Contact Dermatitis*, 18 , 16-19
  DOI: 10.1111/j.1600-0536.1988.tb05483.x
- Foulds IS and Koh D. (1991)
  Contact allergy to 1-acetyl-2-phenylhydrazine in a dimethacrylate adhesive., *Contact Dermatitis*, 25 , 251-252
  DOI: 10.1111/j.1600-0536.1991.tb01854.x
- Suzuki Y and Ohkido M. (1979)
  Contact dermatitis from hydrazine derivatives., *Contact Dermatitis*, 5 , 113-114
  DOI: 10.1111/j.1600-0536.1979.tb04809.x
- Pevny I and Peter G. (1983)
  Allergic contact eczema to pyridine and hydrazine derivatives., *Dermatosen in Beruf und Umwelt*, 31 , 78-83
- National Institute for Occupational Safety and Health (NIOSH). (2014)
  Phenylhydrazine, CAS No. 100-63-0., *National Institute for Occupational Safety and Health (NIOSH) Skin Notation (SK) Profiles*, , 1-24
- European Chemicals Agency (ECHA). (2001)
  Skin sensitisation study for carbonohydrazide (CAS No. 497-18-7)., *European Chemicals Agency Registration Dossier*, ,
- European Chemicals Agency (ECHA). (2004)
  Skin sensitisation study for adipohydrazide (CAS No. 1071-93-8)., *European Chemicals Agency Registration Dossier*, ,

Validation comments

Skin sensitisation: guinea pig maximisation test, local lymph node assay

The alert has demonstrated the following predictive performance:

1) Cronin and Basketter data set: 0 compounds activate this alert
2) Gerberick et al data set: 1 compound activates this alert of which 1 is reported positive (positive predictivity: 100%)
3) Contact Dermatitis data set: 0 compounds activate this alert

1) A collection of guinea pig maximisation test data for 216 compounds from the following reference: Cronin MTD and Basketter DA. Multivariate QSAR analysis of a skin sensitization database. SAR and QSAR in Environmental Research, 1994, 2, 159-179, available at "http://dx.doi.org/10.1080/10629369408029901".

2) A collection of local lymph node assay data for 318 compounds derived from the following references: (i) Gerberick GF, Ryan CA, Kern PS, Schlatter H, Dearman RJ, Kimber I, Patlewicz GY and Basketter DA. Compilation of historical local lymph node data for evaluation of skin sensitization alternative methods. Dermatitis, 2005, 16, 157-202. Downloaded from "http://www.inchemicotox.org/results/" (3 September 2010); (ii) Kern PS, Gerberick GF, Ryan CA, Kimber I, Aptula A and Basketter DA. Local lymph node data for the evaluation of skin sensitization alternatives: a second compilation. Dermatitis, 2010, 21, 8-32, available at "http://dx.doi.org/10.2310/6620.2009.09038".

3) A collection of local lymph node assay data for 137 compounds published in Contact Dermatitis which have been extracted from Vitic Nexus (13 September 2012).

In assessing predictive performance, it should be noted that:

- Mammalian skin sensitisation predictions in Derek associated with a reasoning level of equivocal or above have been considered positive;

- Predictions do not take into account (i) the tautomeric forms of compounds or (ii) the individual components of mixtures;

- Compounds have been considered positive for skin sensitisation if they have been classified as extreme, strong or moderate sensitisers;

- Compounds classified as weak sensitisers have been excluded from the analysis;

- No account has been taken of other skin sensitisation alerts which may also be present in some compounds;

- Information from the data sets may have been used previously as supporting evidence for the derivation of some alerts;

- Some compounds may be present in more than one of the data sets analysed.

EC3 Results

EC3 Result for Derek EC3 Model - 1.2.0

**Species:**	human
**Alert:**	448 Hydrazine or precursor
**Dataset Name:**	Derek EC3 Model - 1.2.0
**Dataset Certified:**	Yes
**Predicted LLNA EC3:**	Insufficient data to make an EC3 Prediction
**Experimental Match:**	No exact match found
**Compounds used in calculation:**	2/2

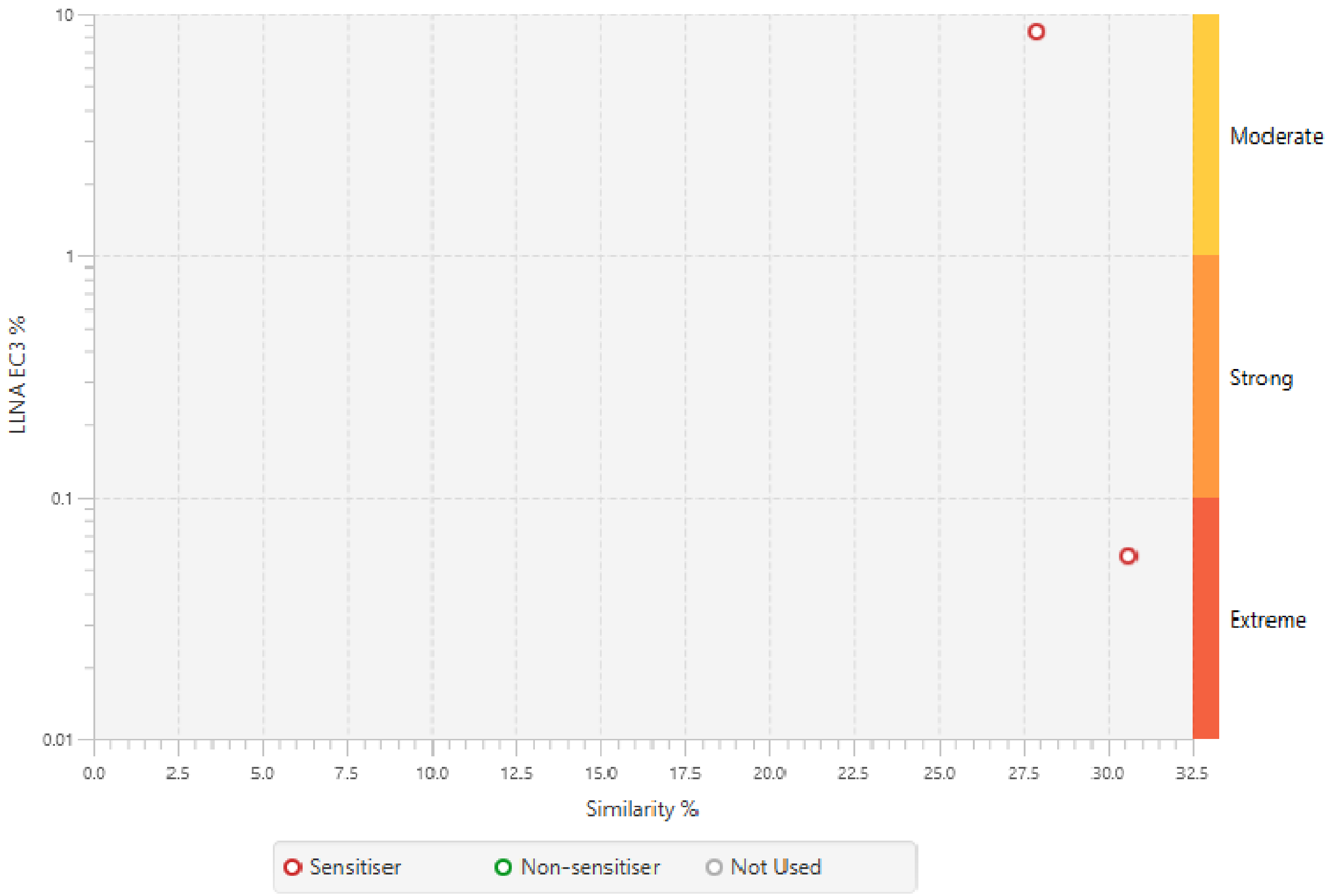

Similar Compounds

Structure

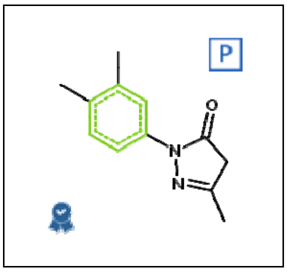

LLNA EC3 % Median: **0.057% (extreme sensitiser)**
Similarity: **31%**
Lhasa Certified: Yes
Used in Calculation: Yes
Number of Studies: 1
Molecular Weight Changed: No
Comments:

Studies

Structure ID	LLNA EC3 % Value	Reference	MWt Changed for Standardisation	Lhasa Certified
Structure-560	0.057	NTP Interagency Center for the Evaluation of Alternative Toxicological Methods (NICEATM), LLNA study for CAS No. 18048-64-1, 2013.	No	Yes

Structure

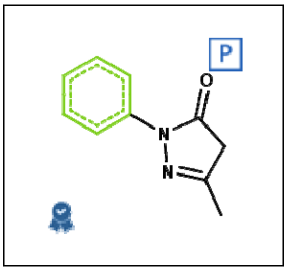

LLNA EC3 % Median: **8.4% (moderate sensitiser)**
Similarity: **28%**
Lhasa Certified: Yes
Used in Calculation: Yes
Number of Studies: 2
Molecular Weight Changed: No
Comments:

Studies

Structure ID	LLNA EC3 % Value	Reference	MWt Changed for Standardisation	Lhasa Certified
Structure-364	8.3	NTP Interagency Center for the Evaluation of Alternative Toxicological Methods (NICEATM), LLNA study for CAS No. 89-25-8, 2013.	No	Yes
Structure-418	8.5	Kern, Dermatitis, 2010, 21, 8-32.	No	Yes

EC3 Results

EC3 Result for Derek EC3 Model - 1.2.0

**Species:**	mammal
**Alert:**	448 Hydrazine or precursor
**Dataset Name:**	Derek EC3 Model - 1.2.0
**Dataset Certified:**	Yes
**Predicted LLNA EC3:**	Insufficient data to make an EC3 Prediction
**Experimental Match:**	No exact match found
**Compounds used in calculation:**	2/2

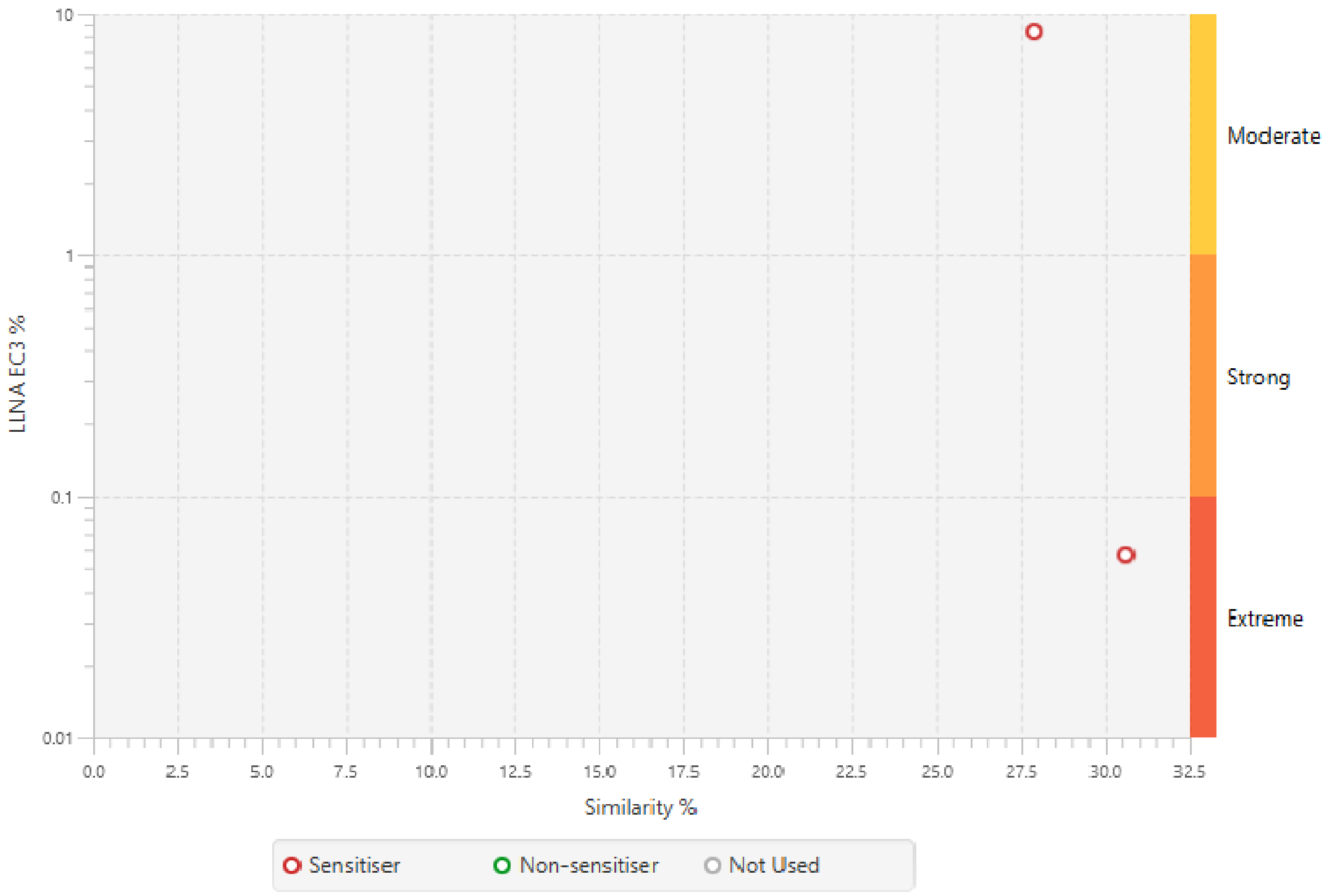

Similar Compounds

Structure

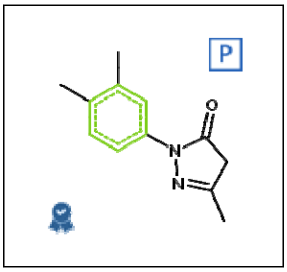

**LLNA EC3 % Median:**	**0.057% (extreme sensitiser)**
**Similarity:**	**31%**
Lhasa Certified:	Yes
Used in Calculation:	Yes
Number of Studies:	1
Molecular Weight Changed:	No
Comments:	

Studies

Structure ID	LLNA EC3 % Value	Reference	MWt Changed for Standardisation	Lhasa Certified
Structure-560	0.057	NTP Interagency Center for the Evaluation of Alternative Toxicological Methods (NICEATM), LLNA study for CAS No. 18048-64-1, 2013.	No	Yes

Structure

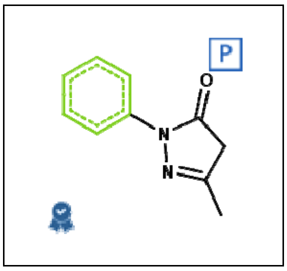

**LLNA EC3 % Median:**	**8.4% (moderate sensitiser)**
**Similarity:**	**28%**
Lhasa Certified:	Yes
Used in Calculation:	Yes
Number of Studies:	2
Molecular Weight Changed:	No
Comments:	

Studies

Structure ID	LLNA EC3 % Value	Reference	MWt Changed for Standardisation	Lhasa Certified
Structure-364	8.3	NTP Interagency Center for the Evaluation of Alternative Toxicological Methods (NICEATM), LLNA study for CAS No. 89-25-8, 2013.	No	Yes
Structure-418	8.5	Kern, Dermatitis, 2010, 21, 8-32.	No	Yes

Examples for Alert 448 Hydrazine or precursor

Example 1: hydrazine

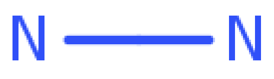

CAS Number: 302-01-2

Test Data (hydrazine)

1)	Species	Assay	Endpoint(s)	Result
	various	various	Skin sensitisation	BgVV category A

References

- Kayser D and Schlede E (editors). (2001)
  Chemikalien und Kontaktallergie: Eine Bewertende Zusammenstellung., *Chemikalien und Kontaktallergie: Eine Bewertende Zusammenstellung*, , All pages

Example 2: phenylhydrazine

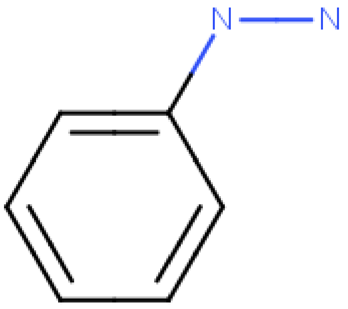

CAS Number: 100-63-0

Example Comments:

Rogan et al tested phenylhydrazine as the HCl salt (CAS 59-88-1) in the Ames test.

Test Data (phenylhydrazine)

1)	Species	Assay	Endpoint(s)	Result
	various	various	Skin sensitisation	BgVV category A

References

- Kayser D and Schlede E (editors). (2001)
  Chemikalien und Kontaktallergie: Eine Bewertende Zusammenstellung., *Chemikalien und Kontaktallergie: Eine Bewertende Zusammenstellung*, , All pages

Alert: 605 Hydrazine (from KB: Derek KB 2018 1.1)

Alert Description Image

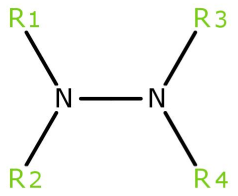

R1-R4 = C, H
At least two of R1-R4 must be H

Match with query compound

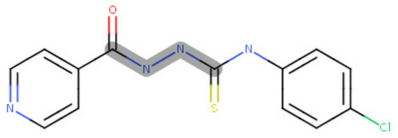

Comments

This alert describes the teratogenicity of primary and secondary hydrazine derivatives.

Hydrazines are used as synthetic intermediates in chemical industry and as therapeutic agents for the treatment of tuberculosis, depression and cancer. Some others are occurring naturally in tobacco, mushrooms, bay leaves and soil [Toth].

The teratogenicity of hydrazines may be due to two distinct mechanism: i) direct binding (e.g. 1,1-dimethylhydrazine and hydrazine) by their free amino group to biomolecules such as Vitamin B6 and ii) generation of reactive free radical species and electrophilic entities (carbonium ions, acylating and diazonium species) by metabolic activation [Kalgutkar et al, ATSDR].

Isoniazid is the only compound studied in humans and it is associated with positive case reports. Intrauterine exposure to isoniazid caused lung anomalies such as mesothelioma and congenital pneumonia and Arthrogryposis Multiplex Congenita (AMC) syndrome, a musculoskeletal disorder characterised by the presence of multiple joint contractures [Tuman et al, Lenke et al].

In the rat, hydrazines caused malformations, foetal and perinatal death and intrauterine growth retardation (IUGR) when given orally (0.31-50 mg/kg/day) or intraperitoneally (2-300 mg/kg/day) [Dluzniewski and Gastol-Lewinska, Chaube and Murphy, Keller et al, Druckrey, Steffek et al, Rao et al] during the organogenesis period (days 6-15 of gestation). Malformations include limb, digital, skeletal and craniofacial defects such as exencephaly, cleft palate and encephalocele. In the rabbit, oral exposure to these chemicals at dose 5 mg/kg/day between days 6-18 of gestation resulted in foetotoxicity and malformations such as skeletal deformities [Dluzniewski and Gastol-Lewinska, Harper and Worden, Mercier-Parot and Tuchmann-Duplessis].

The scope of the alert is based on the structural features able to generate active radical species or electrophilic entities [Kalgutkar et al].

References

- Kalgutkar AS, Gardner I, Obach RS, Shaffer CL, Callegari E, Henne KR, Mutlib AE, Dalvie DK, Lee JS, Nakai Y, O'Donnell JP, Boer J and Harriman SP. (2005)
  A comprehensive listing of bioactivation pathways of organic functional groups., *Current Drug Metabolism*, 6 , 161-225
  DOI: 10.2174/1389200054021799

- Chaube S and Murphy ML. (1968)
  The teratogenic effects of the recent drugs active in cancer chemotherapy., *Advances in Teratology*, 3 , 181-237

- Lenke RR,Turkel SB and Monsen R. (1985)
  Severe fetal deformities associated with ingestion of excessive isoniazid in early pregnancy., *Acta Obstetricia et Gynecologica Scandinavica*, 64 , 281-282

- Tuman KJ, Chilcote RR, Berkow RI and Moohr JW. (1980)
  Mesothelioma in child with prenatal exposure to isoniazid., *The Lancet*, 2 , 362

- Dluzniewski A and Gastol-Lewinska L. (1971)
  The search for teratogenic activity of some tuberculostatic drugs., *Dissertationes Pharmaceuticae et Pharmacologicae*, 23 , 383-392

- Steffek AJ, Verrusio AC and Watkins CA. (1972)
  Cleft palate in rodents after maternal treatment with various lathyrogenic agents., *Teratology*, 5 , 33-40
  DOI: 10.1002/tera.1420050107
- Toth B. (1993)
  Teratogenic hydrazines: a review., *In Vivo*, 7 , 101-110

- Agency for Toxic Substances and Disease Registry (ATSDR). (1997)
  Toxicological profile for hydrazines. 2. Health effects., *Agency for Toxic Substances and Disease Registry Report*, , 11-107

- Keller WC, Olson CT, Back KC and Gaworski CL. (1984)
  Teratogenic assessment of three methylated hydrazine derivatives in the rat., *Journal of Toxicology and Environmental Health*, 13 , 125-131

- Druckrey H. (1973)
  Specific carcinogenic and teratogenic effects of 'indirect' alkylating methyl and ethyl compounds and their dependency on stages on ontogenic developments., *Xenobiotica*, 3 , 271-303

- Rao RR, Bhat NG, Nair TB and Shukla RG. (1973)
  Toxicologic and teratologic studies with 1-[(3,5-bis-trifluoromethyl)phenyl]-4-methyl-thiosemicarbazide (CIBA 2696Go)., *Arzneimittel-Forschung*, 23 , 797-800

- Harper KH and Worden AN. (1966)
  Comparative toxicity of isonicotinic acid hydrazide and its methansulfonate derivative., *Toxicology and Applied Pharmacology*, 8 , 325-333
  DOI: 10.1016/S0041-008X(66)80019-4
- Mercier-Parot L and Tuchmann-Duplessis H. (1969)
  Embryotoxic and teratogenic action of a methylhydrazine in mice and rabbits (French)., *Comptes Rendus des Seances de la Societe de Biologie et de ses Filiales*, 163 , 16-20

Examples for Alert 605 Hydrazine

Example 1: semicarbazide

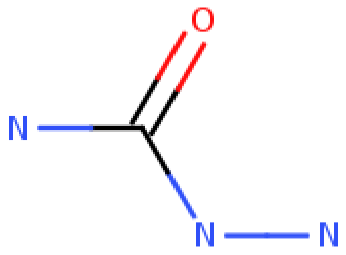

CAS Number: 57-56-7

Test Data (semicarbazide)

	Species	Assay	Endpoint(s)	Result
**1)**	rat	teratogenicity study	Teratogenicity	positive

References

- Steffek AJ, Verrusio AC and Watkins CA. (1972)
  Cleft palate in rodents after maternal treatment with various lathyrogenic agents., *Teratology*, 5 , 33-40
  DOI: 10.1002/tera.1420050107

Alert: 615 Hydrazine (from KB: Derek KB 2018 1.1)

Alert Description Image

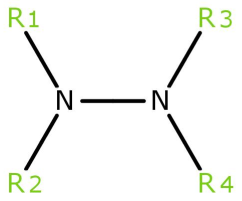

Match with query compound

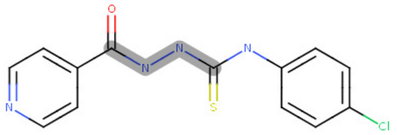

R1-R4 = H, C
At least two R groups must be H

Comments

This alert describes the hepatotoxicity of hydrazine derivatives. These compounds are known to cause a hepatocellular type injury both in experimental animals (necrosis and/or steatosis) and in humans (necrosis).

Hydrazine derivatives are used as synthetic intermediates in the chemical industry, and as therapeutic agents for the treatment of tuberculosis, depression and cancer [Zimmerman, Hussain and Frazier].

Typical hepatotoxins include hydrazine itself, isoniazid (INH), iproniazid and hydralazine. The injury has been reflected by significant elevations in aminotransferase levels and/or necrosis [Zimmerman, Black and Hussain]. Hepatic steatosis subsequent to occupational exposure to hydrazines has also been described [Zimmerman]. Age and the use of P450 inducers such as alcohol are thought to enhance susceptibility whereas the role of genetic polymorphism remains controversial [Zimmerman]. In general, no dose-response relationship has been established. Iproniazid has been withdrawn from use because of a high incidence of case reports [Zimmerman, Kahn and Perez]. INH has been associated with biochemical evidence of hepatic injury and overt hepatitis in up to 20% and 2% of recipients respectively [Anonymous, Zimmerman, Black et al]. Hydralazine has caused additional hepatic dysfunctions in the form of granulomas, cholangitis and cholestasis [Zimmerman, Myers and Augur]. Clinical signs of hypersensitivity (fever, rash, eosinophilia, positive rechallenge with the offending drug and detection of antibodies) have also been observed with hydralazine [Zimmerman].

Steatosis and/or necrosis have been reproduced in several species with hydrazine [Patrick and Back, Swiecicki et al, Richards et al, Yard and Mckennis], in rats with iproniazid [Nelson et al] and mainly in rabbits with related compounds [Patrick and Back, Nelson et al, Yard and Mckennis]. Additionally, some derivatives have been associated with apparent hepatocarcinogenicity [Zimmerman].

Hydrazine-induced hepatotoxicity has been proven to require metabolic activation by the cytochrome P450 system [Timbrell, Kalgutkar et al]. The proposed mechanism involves the monosubstituted hydrazine moiety borne by or derived from the parent compound. Following N-hydroxylation and spontaneous hydrolysis, a diazine intermediate is generated. The latter may undergo homolytic cleavage or an oxidation process leading to free radicals or electrophilic entities (carbonium ions, acylating and diazonium species) [Timbrell, Kalgutkar et al]. Alternatively, a mechanism that could be mediated by the release of reactive oxygen species and subsequent oxidative stress has been suggested [Hussain and Frazier].

The scope of the alert is based on i) the chemical features shared by the compounds discussed, ii) some consideration of potential precursors (hydrolysis of N-acetyl groups or oxidative N-dealkylation) and iii) the proposed mechanism that requires a monosubstituted hydrazine moiety.

References

- Anonymous. (2005)
  Physicians' Desk Reference (PDR)., *Physicians' Desk Reference*, , All pages

- Zimmerman HJ. (1999)
  Hepatotoxicity: The Adverse Effects of Drugs and other Chemicals on the Liver., *Hepatotoxicity: The Adverse Effects of Drugs and other Chemicals on the Liver*, , All pages

- Kalgutkar AS, Gardner I, Obach RS, Shaffer CL, Callegari E, Henne KR, Mutlib AE, Dalvie DK, Lee JS, Nakai Y, O'Donnell JP, Boer J and Harriman SP. (2005)
  A comprehensive listing of bioactivation pathways of organic functional groups., *Current Drug Metabolism*, 6 , 161-225
  DOI: 10.2174/1389200054021799
- Swiecicki W, Kwarecki K, Rozynski J and Sarol Z. (1973)
  Morphological and biochemical investigations on chronic poisoning with hydrazine hydrate in guinea pigs (Polish)., *Acta Poloniae Pharmaceutica*, 30 , 213-221

- Richards VE, Chau B, White MR and McQueen CA. (2004)
  Hepatic gene expression and lipid homeostasis in C57BL/6 mice exposed to hydrazine or acetylhydrazine., *Toxicological Sciences*, 82 , 318-332
  DOI: 10.1093/toxsci/kfh232

- Black M and Hussain H. (2000)
  Hydrazine, cancer, the internet, isoniazid, and the liver., *Annals of Internal Medicine*, 133 , 911-913

- Black M, Mitchell JR, Zimmerman HJ, Ishak KG and Epler GR. (1975)
  Isoniazid-associated hepatitis in 114 patients., *Gastroenterology*, 69 , 289-302

- Hussain SM and Frazier JM. (2002)
  Cellular toxicity of hydrazine in primary rat hepatocytes., *Toxicological Sciences*, 69 , 424-432
  DOI: 10.1093/toxsci/69.2.424
- Kahn M and Perez V. (1958)
  Jaundice associated with the administration of iproniazid; report of nine cases., *American Journal of Medicine*, 25 , 898-916
  DOI: 10.1016/0002-9343(58)90062-7
- Myers JL and Augur NA Jr. (1984)
  Hydralazine-induced cholangitis., *Gastroenterology*, 87 , 1185-1188

- Nelson SD, Mitchell JR, Snodgrass WR and Timbrell JA. (1978)
  Hepatotoxicity and metabolism of iproniazid and isopropylhydrazine., *Journal of Pharmacology and Experimental Therapeutics*, 206 , 574-585

- Patrick RL and Back KC. (1965)
  Pathology and toxicology of repeated doses of hydrazine and 1,1-dimethylhydrazine in monkeys and rats., *Industrial Medicine and Surgery*, 34 , 430-435

- Timbrell JA. (1979)
  The role of metabolism in the hepatotoxicity of isoniazid and iproniazid., *Drug Metabolism Reviews*, 10 , 125-147
  DOI: 10.3109/03602537908993904
- Yard AS and McKennis H Jr. (1955)
  Effect of structure on the ability of hydrazino compounds to produce fatty livers., *Journal of Pharmacology and Experimental Therapeutics*, 114 , 391-397

Examples for Alert 615 Hydrazine

Example 1: hydrazine

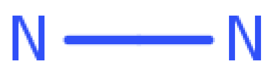

CAS Number: 302-01-2

Test Data (hydrazine)

1)

Species	Assay	Endpoint(s)	Result
human	hepatotoxicity case study	Hepatotoxicity	positive

References
- Black M and Hussain H. (2000)
  Hydrazine, cancer, the internet, isoniazid, and the liver., *Annals of Internal Medicine*, 133 , 911-913

2)

Species	Assay	Endpoint(s)	Result
rabbit	hepatotoxicity study	Hepatotoxicity	positive

References
- Yard AS and McKennis H Jr. (1955)
  Effect of structure on the ability of hydrazino compounds to produce fatty livers., *Journal of Pharmacology and Experimental Therapeutics*, 114 , 391-397

3)

Species	Assay	Endpoint(s)	Result
rat	hepatotoxicity study	Hepatotoxicity	positive

References
- Patrick RL and Back KC. (1965)
  Pathology and toxicology of repeated doses of hydrazine and 1,1-dimethylhydrazine in monkeys and rats., *Industrial Medicine and Surgery*, 34 , 430-435

4)

Species	Assay	Endpoint(s)	Result
mouse	hepatotoxicity study	Hepatotoxicity	positive

References
- Richards VE, Chau B, White MR and McQueen CA. (2004)
  Hepatic gene expression and lipid homeostasis in C57BL/6 mice exposed to hydrazine or acetylhydrazine., *Toxicological Sciences*, 82 , 318-332
  DOI: 10.1093/toxsci/kfh232

5)

Species	Assay	Endpoint(s)	Result

guinea pig	hepatotoxicity study	Hepatotoxicity	positive

References

- Swiecicki W, Kwarecki K, Rozynski J and Sarol Z. (1973)
  Morphological and biochemical investigations on chronic poisoning with hydrazine hydrate in guinea pigs (Polish)., *Acta Poloniae Pharmaceutica*, 30 , 213-221

6)	Species	Assay	Endpoint(s)	Result
	monkey	hepatotoxicity study	Hepatotoxicity	positive

References

- Patrick RL and Back KC. (1965)
  Pathology and toxicology of repeated doses of hydrazine and 1,1-dimethylhydrazine in monkeys and rats., *Industrial Medicine and Surgery*, 34 , 430-435

Example 2: isoniazid

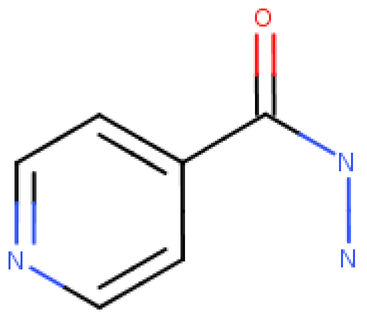

CAS Number: 54-85-3

Test Data (isoniazid)

1)	Species	Assay	Endpoint(s)	Result
	human	hepatotoxicity case study	Hepatotoxicity	positive

References

- Black M, Mitchell JR, Zimmerman HJ, Ishak KG and Epler GR. (1975)
  Isoniazid-associated hepatitis in 114 patients., *Gastroenterology*, 69 , 289-302

- Anonymous. (2005)
  Physicians' Desk Reference (PDR)., *Physicians' Desk Reference*, , All pages

Example 3: iproniazid

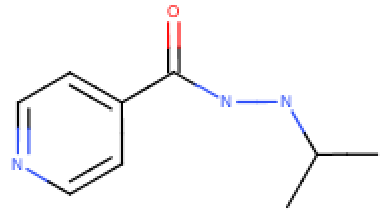

CAS Number: 54-92-2

Test Data (iproniazid)

	Species	Assay	Endpoint(s)	Result
1)	human	hepatotoxicity case report	Hepatotoxicity	positive

References

- Kahn M and Perez V. (1958)
  Jaundice associated with the administration of iproniazid; report of nine cases., *American Journal of Medicine*, 25 , 898-916
  DOI: 10.1016/0002-9343(58)90062-7

	Species	Assay	Endpoint(s)	Result
2)	rat	hepatotoxicity study	Hepatotoxicity	positive

References

- Timbrell JA. (1979)
  The role of metabolism in the hepatotoxicity of isoniazid and iproniazid., *Drug Metabolism Reviews*, 10 , 125-147
  DOI: 10.3109/03602537908993904
- Nelson SD, Mitchell JR, Snodgrass WR and Timbrell JA. (1978)
  Hepatotoxicity and metabolism of iproniazid and isopropylhydrazine., *Journal of Pharmacology and Experimental Therapeutics*, 206 , 574-585

Example 4: hydralazine

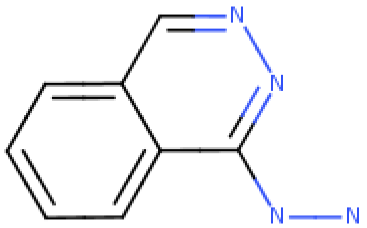

CAS Number: 86-54-4

Test Data (hydralazine)

	Species	Assay	Endpoint(s)	Result
1)	human	hepatotoxicity case report	Hepatotoxicity	positive

References

- Myers JL and Augur NA Jr. (1984)
  Hydralazine-induced cholangitis., *Gastroenterology*, 87 , 1185-1188

2)	Species	Assay	Endpoint(s)	Result
	human	hepatotoxicity case study	Hepatotoxicity	positive

References

- Zimmerman HJ. (1999)
  Hepatotoxicity: The Adverse Effects of Drugs and other Chemicals on the Liver., *Hepatotoxicity: The Adverse Effects of Drugs and other Chemicals on the Liver*, , All pages

Alert: RapidPrototype035 Halogenated benzene (from KB: Derek KB 2018 1.1)

Alert Description Image	Match with query compound

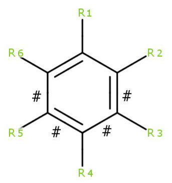

R1 = F, Cl, Br
R2-R6 = H, C% (alkyl), OH, OC@, N£, F, Cl, Br
Bonds marked # are not allowed to be fused
Atoms marked % cannot be bound to more than one heteroatom
Atoms marked @ cannot be aromatic or bound to any heteroatoms
Atoms marked £ cannot be aromatic, doubly bonded or bound to any heteroatoms

Pentafluoro derivatives have been excluded

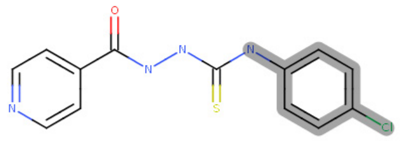

Comments

This alert describes the nephrotoxicity of halogenated benzenes. This is a rapid prototype alert derived using a proprietary data set of 731 chemicals, classified on the basis of the presence or absence of histopathologic lesions in the kidney in oral rat repeat dose studies mostly of 28-days duration. Forty-two chemicals in this data set activated this rapid prototype alert and twenty-two of these were nephrotoxic.

Alert: RapidPrototype036 Hydrazine (from KB: Derek KB 2018 1.1)

Alert Description Image

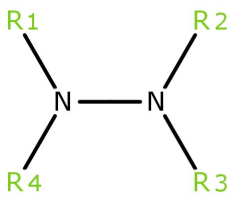

R1-R4 = H, C
At least two R groups must be H

Match with query compound

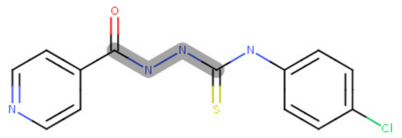

Comments

This alert describes the nephrotoxicity of hydrazines. This is a rapid prototype alert derived using a proprietary data set of 731 chemicals, classified on the basis of the presence or absence of histopathologic lesions in the kidney in oral rat repeat dose studies mostly of 28-days duration. Six chemicals in this data set activated this rapid prototype alert and four of these were nephrotoxic.

Alert: RapidPrototype108 Hydrazine (from KB: Derek KB 2018 1.1)

Alert Description Image | **Match with query compound**

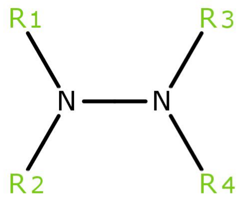

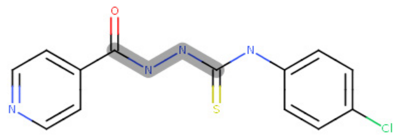

R1-R4 = H, C
At least two R groups must be H

Comments

This alert describes the mitochondrial dysfunction caused by hydrazines. This is a rapid prototype alert derived using a proprietary data set of 362 compounds and two published data sets of 105 [Mehta et al] (data set A) and 283 compounds [Zhang et al] (data set B) respectively. Compounds were classified on the basis of their uncoupling/inhibitory activity on oxidative phosphorylation (proprietary data set) or their reported effects on mitochondria in the published literature (published data sets A and B).

1) Proprietary data set: 3 compounds activate this rapid prototype alert of which 0 are reported positive

2) Published data set A: 2 compounds activate this rapid prototype alert of which 2 are reported positive

3) Published data set B: 1 compound activates this rapid prototype alert of which 1 is reported positive

References

- Mehta R, Chan K, Lee O, Tafazoli S and O'Brien PJ. (2008)
  Drug-associated mitochondrial toxicity., *Drug-Induced Mitochondrial Dysfunction*, , 71-126

- Zhang H, Chen QY, Xiang ML, Ma CY, Huang Q and Yang SY. (2009)
  In silico prediction of mitochondrial toxicity by using GA-CG-SVM approach., *Toxicology in Vitro*, 23 , 134-140
  DOI: 10.1016/j.tiv.2008.09.017

Reasoning Details

Carcinogenicity in human is PLAUSIBLE (from KB: Derek KB 2018 1.1)

The parameters that have influenced your prediction are: substructures in the input structure, which have the potential to cause carcinogenicity; your selected species, which is human.

Rule 188: If [alert 114] is [certain] then [Carcinogenicity alert set 1] is [certain]
- alert 114 **is** CERTAIN

If a chemical contains alert 114 then it is a member of "Carcinogenicity alert set 1". Membership of "Carcinogenicity alert set 1" indicates the presence of a carcinogenicity alert associated with a genotoxic mechanism of action.

Rule 243: If [species mammal] is [certain] then [Species dependent variable 22] is [plausible]
- species mammal **is** CERTAIN

In mammals the variable "Species dependent variable 22" is plausible.

Rule 210: If [Carcinogenicity alert set 1] is [certain] then [Carcinogenicity] is [Species dependent variable 22]
- Carcinogenicity alert set 1 **is** CERTAIN
- Species dependent variable 22 **is** PLAUSIBLE

If a chemical is a member of "Carcinogenicity alert set 1" then it is considered plausible that the chemical will cause carcinogenicity in mammals and impossible in bacteria. The variation in rule outcome with species is achieved via use of the variable "Species dependent variable 22".

Carcinogenicity in mammal is PLAUSIBLE (from KB: Derek KB 2018 1.1)

The parameters that have influenced your prediction are: substructures in the input structure, which have the potential to cause carcinogenicity; your selected species, which is mammal.

Rule 188: If [alert 114] is [certain] then [Carcinogenicity alert set 1] is [certain]
- alert 114 **is** CERTAIN

If a chemical contains alert 114 then it is a member of "Carcinogenicity alert set 1". Membership of "Carcinogenicity alert set 1" indicates the presence of a carcinogenicity alert associated with a genotoxic mechanism of action.

Rule 243: If [species mammal] is [certain] then [Species dependent variable 22] is [plausible]
- species mammal **is** CERTAIN

In mammals the variable "Species dependent variable 22" is plausible.

Rule 210: If [Carcinogenicity alert set 1] is [certain] then [Carcinogenicity] is [Species dependent variable 22]
- Carcinogenicity alert set 1 **is** CERTAIN
- Species dependent variable 22 **is** PLAUSIBLE

If a chemical is a member of "Carcinogenicity alert set 1" then it is considered plausible that the chemical will cause carcinogenicity in mammals and impossible in bacteria. The variation in rule outcome with species is achieved via use of the variable "Species dependent variable 22".

Hepatotoxicity in human is PLAUSIBLE (from KB: Derek KB 2018 1.1)

The parameters that have influenced your prediction are: substructures in the input structure, which have the potential to cause hepatotoxicity; your selected species, which is human.

Rule 243: If [species mammal] is [certain] then [Species dependent variable 22] is [plausible]
- species mammal **is** CERTAIN

In mammals the variable "Species dependent variable 22" is plausible.

Rule 1292: If [alert 615] is [certain] then [Hepatotoxicity] is [Species dependent variable 22]
- alert 615 **is** CERTAIN
- Species dependent variable 22 **is** PLAUSIBLE

If a chemical contains alert 615 then it is considered plausible that the chemical will cause hepatotoxicity in mammals and impossible in bacteria. The variation in rule outcome with species is achieved via use of the variable "Species dependent variable 22".

Hepatotoxicity in mammal is PLAUSIBLE (from KB: Derek KB 2018 1.1)

The parameters that have influenced your prediction are: substructures in the input structure, which have the potential to cause hepatotoxicity; your selected species, which is mammal.

Rule 243: If [species mammal] is [certain] then [Species dependent variable 22] is [plausible]
- species mammal **is** CERTAIN

In mammals the variable "Species dependent variable 22" is plausible.

Rule 1292: If [alert 615] is [certain] then [Hepatotoxicity] is [Species dependent variable 22]
- alert 615 **is** CERTAIN
- Species dependent variable 22 **is** PLAUSIBLE

If a chemical contains alert 615 then it is considered plausible that the chemical will cause hepatotoxicity in mammals and impossible in bacteria. The variation in rule outcome with species is achieved via use of the variable "Species dependent variable 22".

Mitochondrial dysfunction in human is EQUIVOCAL (from KB: Derek KB 2018 1.1)

The parameters that have influenced your prediction are: substructures in the input structure, which have the potential to cause mitochondrial dysfunction; your selected species, which is human.

Rule 266: If [species mammal] is [certain] then [Species dependent variable 8] is [equivocal]

- species mammal **is** CERTAIN

In mammals the variable "Species dependent variable 8" is equivocal.

Rule 1033: If [Mitochondrial dysfunction alert] is [certain] then [Mitochondrial dysfunction] is [Species dependent variable 8]

- Mitochondrial dysfunction alert **is** CERTAIN
- Species dependent variable 8 **is** EQUIVOCAL

If a chemical contains a rapid prototype alert for mitochondrial dysfunction then it is considered equivocal that the chemical will cause mitochondrial dysfunction in mammals and impossible in bacteria. The variation in rule outcome with species is activated via use of the variable "Species dependent variable 8".

Mitochondrial dysfunction in mammal is EQUIVOCAL (from KB: Derek KB 2018 1.1)

The parameters that have influenced your prediction are: substructures in the input structure, which have the potential to cause mitochondrial dysfunction; your selected species, which is mammal.

Rule 266: If [species mammal] is [certain] then [Species dependent variable 8] is [equivocal]

- species mammal **is** CERTAIN

In mammals the variable "Species dependent variable 8" is equivocal.

Rule 1033: If [Mitochondrial dysfunction alert] is [certain] then [Mitochondrial dysfunction] is [Species dependent variable 8]

- Mitochondrial dysfunction alert **is** CERTAIN
- Species dependent variable 8 **is** EQUIVOCAL

If a chemical contains a rapid prototype alert for mitochondrial dysfunction then it is considered equivocal that the chemical will cause mitochondrial dysfunction in mammals and impossible in bacteria. The variation in rule outcome with species is activated via use of the variable "Species dependent variable 8".

Mutagenicity in vitro in bacterium is INACTIVE (from KB: Derek KB 2018 1.1)

The parameters that have influenced your prediction are: substructures in the input structure, which have the potential to cause mutagenicity; your selected species, which is bacterium.

Overview
Contains unclassified features

Unclassified features

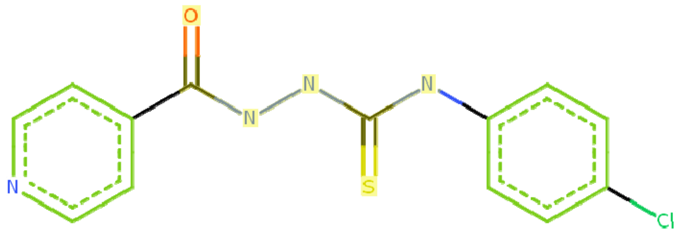

Details
The query structure contains features (highlighted in the structure panel) that were not found in the Lhasa Ames test reference set and do not match any structural alerts or examples for (bacterial in vitro) mutagenicity in Derek. It is predicted to be inactive in the bacterial in vitro (Ames) mutagenicity test.

Nephrotoxicity in human is EQUIVOCAL (from KB: Derek KB 2018 1.1)

The parameters that have influenced your prediction are: substructures in the input structure, which have the potential to cause nephrotoxicity; your selected species, which is human.

Rule 266: If [species mammal] is [certain] then [Species dependent variable 8] is [equivocal]
- species mammal **is** CERTAIN

In mammals the variable "Species dependent variable 8" is equivocal.

Rule 995: If [alert RapidPrototype035] is [certain] then [Nephrotoxicity] is [Species dependent variable 8]
- alert RapidPrototype035 **is** CERTAIN
- Species dependent variable 8 **is** EQUIVOCAL

If a chemical contains rapid prototype alert 035 then it is considered equivocal that the chemical will cause nephrotoxicity in mammals and impossible in bacteria. The variation in rule outcome with species is achieved via use of the variable "Species dependent variable 8".

Rule 266: If [species mammal] is [certain] then [Species dependent variable 8] is [equivocal]
- species mammal **is** CERTAIN

In mammals the variable "Species dependent variable 8" is equivocal.

Rule 1201: If [alert RapidPrototype036] is [certain] then [Nephrotoxicity] is [Species dependent variable 8]
- alert RapidPrototype036 **is** CERTAIN
- Species dependent variable 8 **is** EQUIVOCAL

If a chemical contains rapid prototype alert 036 then it is considered equivocal that the chemical will cause nephrotoxicity in mammals and impossible in bacteria. The variation in rule outcome with species is achieved via use of the variable "Species dependent variable 8".

Nephrotoxicity in mammal is EQUIVOCAL (from KB: Derek KB 2018 1.1)

The parameters that have influenced your prediction are: substructures in the input structure, which have the potential to cause nephrotoxicity; your selected species, which is mammal.

Rule 266: If [species mammal] is [certain] then [Species dependent variable 8] is [equivocal]
- species mammal **is** CERTAIN

In mammals the variable "Species dependent variable 8" is equivocal.

Rule 995: If [alert RapidPrototype035] is [certain] then [Nephrotoxicity] is [Species dependent variable 8]
- alert RapidPrototype035 **is** CERTAIN
- Species dependent variable 8 **is** EQUIVOCAL

If a chemical contains rapid prototype alert 035 then it is considered equivocal that the chemical will cause nephrotoxicity in mammals and impossible in bacteria. The variation in rule outcome with species is achieved via use of the variable "Species dependent variable 8".

Rule 266: If [species mammal] is [certain] then [Species dependent variable 8] is [equivocal]
- species mammal **is** CERTAIN

In mammals the variable "Species dependent variable 8" is equivocal.

Rule 1201: If [alert RapidPrototype036] is [certain] then [Nephrotoxicity] is [Species dependent variable 8]
- alert RapidPrototype036 **is** CERTAIN
- Species dependent variable 8 **is** EQUIVOCAL

If a chemical contains rapid prototype alert 036 then it is considered equivocal that the chemical will cause nephrotoxicity in mammals and impossible in bacteria. The variation in rule outcome with species is achieved via use of the variable "Species dependent variable 8".

Skin sensitisation in human is EQUIVOCAL (from KB: Derek KB 2018 1.1)

The parameters that have influenced your prediction are: substructures in the input structure, which have the potential to cause skin sensitisation; your selected species, which is human.

Rule 266: If [species mammal] is [certain] then [Species dependent variable 8] is [equivocal]
- species mammal **is** CERTAIN

In mammals the variable "Species dependent variable 8" is equivocal.

Rule 923: If [alert 448] is [certain] then [Skin sensitisation] is [Species dependent variable 8]
- alert 448 **is** CERTAIN
- Species dependent variable 8 **is** EQUIVOCAL

If a chemical contains alert 448 then it is considered equivocal that the chemical will cause skin sensitisation in mammals and impossible in bacteria. The variation in rule outcome with species is achieved via use of the variable "Species dependent variable 8".

Skin sensitisation in mammal is EQUIVOCAL (from KB: Derek KB 2018 1.1)

The parameters that have influenced your prediction are: substructures in the input structure, which have the potential to cause skin sensitisation; your selected species, which is mammal.

Rule 266: If [species mammal] is [certain] then [Species dependent variable 8] is [equivocal]
- species mammal **is** CERTAIN

In mammals the variable "Species dependent variable 8" is equivocal.

Rule 923: If [alert 448] is [certain] then [Skin sensitisation] is [Species dependent variable 8]
- alert 448 **is** CERTAIN
- Species dependent variable 8 **is** EQUIVOCAL

If a chemical contains alert 448 then it is considered equivocal that the chemical will cause skin sensitisation in mammals and impossible in bacteria. The variation in rule outcome with species is achieved via use of the variable "Species dependent variable 8".

Teratogenicity in human is PLAUSIBLE (from KB: Derek KB 2018 1.1)

The parameters that have influenced your prediction are: substructures in the input structure, which have the potential to cause teratogenicity; your selected species, which is human.

Rule 243: If [species mammal] is [certain] then [Species dependent variable 22] is [plausible]
- species mammal **is** CERTAIN

In mammals the variable "Species dependent variable 22" is plausible.

Rule 1156: If [alert 605] is [certain] then [Teratogenicity] is [Species dependent variable 22]
- alert 605 **is** CERTAIN
- Species dependent variable 22 **is** PLAUSIBLE

If a chemical contains alert 605 then it is considered plausible that the chemical will cause teratogenicity in mammals and impossible in bacteria. The variation in rule outcome with species is achieved via use of the variable "Species dependent variable 22".

Teratogenicity in mammal is PLAUSIBLE (from KB: Derek KB 2018 1.1)

The parameters that have influenced your prediction are: substructures in the input structure, which have the potential to cause teratogenicity; your selected species, which is mammal.

Rule 243: If [species mammal] is [certain] then [Species dependent variable 22] is [plausible]

- species mammal **is** CERTAIN

In mammals the variable "Species dependent variable 22" is plausible.

Rule 1156: If [alert 605] is [certain] then [Teratogenicity] is [Species dependent variable 22]

- alert 605 **is** CERTAIN
- Species dependent variable 22 **is** PLAUSIBLE

If a chemical contains alert 605 then it is considered plausible that the chemical will cause teratogenicity in mammals and impossible in bacteria. The variation in rule outcome with species is achieved via use of the variable "Species dependent variable 22".

Glossary

Certain
There is proof that the proposition is true.

Probable
There is at least one strong argument that the proposition is true and there are no arguments against it.

Plausible
The weight of evidence supports the proposition.

Equivocal
There is an equal weight of evidence for and against the proposition.

Doubted
The weight of evidence opposes the proposition.

Improbable
There is at least one strong argument that the proposition is false and there are no arguments that it is true.

Impossible
There is proof that the proposition is false.

Open
There is no evidence that supports or opposes the proposition.

Contradicted
There is proof that the proposition is both true and false.

Inactive, no misclassified or unclassified features
The query structure does not match any structural alerts or examples in Derek which show activity in a bacterial reverse mutation assay (Ames test). Additionally, the query structure does not contain any unclassified or misclassified features.

Inactive, contains misclassified features
Features in the molecule are found in non-alerting mutagens in the Lhasa reference set. The prediction remains negative and the misclassified features are highlighted to enable the negative prediction to be verified by expert assessment.

Inactive, contains unclassified features
Some features in the molecule have not been found in the Lhasa reference set. The prediction remains negative and the unclassified features are highlighted to enable the negative prediction to be verified by expert assessment.

Inactive, contains misclassified and unclassified features
The query structure contains features that are misclassified and features that are unclassified. These are highlighted on the structure.

Non-sensitiser, no misclassified or unclassified features
The query structure does not match any structural alerts or examples for skin sensitisation in Derek. Additionally, the query structure does not contain any unclassified or misclassified features.

Non-sensitiser, contains misclassified features
Features in the molecule are found in non-alerting sensitisers in the Lhasa skin sensitisation negative prediction dataset. The prediction remains negative and the misclassified features are highlighted to enable the negative prediction to be verified by expert assessment.

Non-sensitiser, contains unclassified features
Some features in the molecule have not been found in the Lhasa skin sensitisation negative prediction dataset. The prediction remains negative and the unclassified features are highlighted to enable the negative prediction to be verified by expert assessment.

Non-sensitiser, contains misclassified and unclassified features
The query structure contains features that are misclassified and features that are unclassified. These are highlighted on the structure.
