# Supplementary material for: Toxicity Study and Binding Analysis of Newly Synthesized Antifungal N-(4-aryl/cyclohexyl)-2-(pyridine-4-yl carbonyl) hydrazinecarbothioamide Derivative with Bovine Serum Albumin
Source: Int J Mol Sci. 2023 Mar 3;24(5):4942. doi: 10.3390/ijms24054942 (PMC10002925; doi:10.3390/ijms24054942)
Supplement: Supplementary file 1 [file ijms-24-04942-s001.zip › Supplementary File S2.doc.pdf]

Toxicity Study and Binding Analysis of Newly Synthesized Antifungal *N*-(4-aryl/cyclohexyl)-2-(pyridine-4-yl carbonyl) hydrazinecarbothioamide Derivative with Bovine Serum Albumin

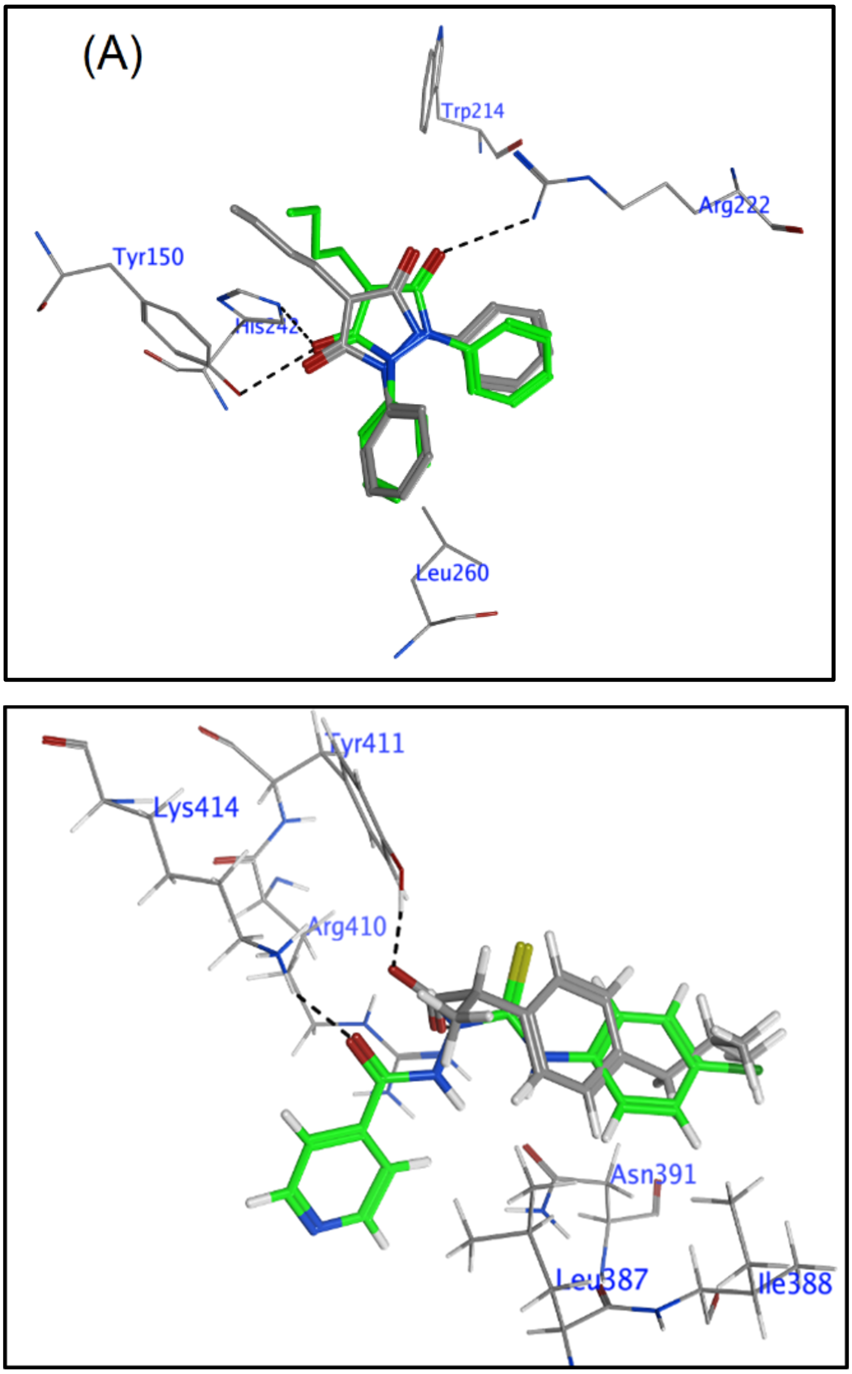

Figure S1. (**A**) Docking Validation of site I in HSA (PDB ID: 2BXC) using phenylbutazone as co-crystallized ligand; (**B**) Docking Validation of site II in HSA (PDB ID: 2BXG) using ibuprofen as co-crystallized ligand.
